# A Computational Strategy for Identifying Self‐Assembling Food‐Derived Molecules for Antiparasitic Nanotherapy

**DOI:** 10.1002/advs.202524297

**Published:** 2026-05-29

**Authors:** Shenye Qu, Ting Wang, Jietao Liu, Jiacheng Qin, Bin Yang, Yihang Liu, Pengfei Li, Gaoxue Wang, Fei Ling

**Affiliations:** ^1^ College of Animal‐Science and Technology Northwest A&F University Yangling Shaanxi China; ^2^ College of Enology Northwest A&F University Yangling Shaanxi China; ^3^ Guangxi Academy of Sciences Nanning China

**Keywords:** antiparasitic nanotherapy, computational screening, carrier‐free nanodrugs, food‐derived molecules, self‐assembly

## Abstract

Carrier‐free nanodrugs remain difficult to design, and the molecular basis of their self‐assembly is still poorly understood. Here, an integrated workflow combining 2D/3D molecular screening with SHAP‐assisted analysis was used to identify self‐assembling pairs from food‐derived compounds. Ursolic acid and 18*β*‐glycyrrhetinic acid were thereby identified and found to self‐assemble into stable nanoparticles (UA‐18*β*GA NPs). Compared with the individual components, nanoparticles showed reduced cytotoxicity and enhanced antiparasitic activity under the tested conditions. Spectroscopic characterization together with density functional theory calculations and molecular dynamics simulations supported the intermolecular interactions and structural evolution associated with nanoparticle formation. UA‐18*β*GA NPs exhibited synergistic antiparasitic activity against *Ichthyophthirius multifiliis*, while also showing reduced combined toxicity relative to the free components. Mechanistically, the nanoparticles were associated with parasite apoptosis involving Erk1/Akt‐related signaling. In infected zebrafish, UA‐18*β*GA NPs reduced oxidative stress and inflammatory responses, accompanied by altered macrophage marker expression and reduced inflammasome‐related gene expression. In a murine model of experimental cerebral malaria, the nanoparticles improved therapeutic outcomes, reduced blood‐brain barrier leakage, and attenuated inflammatory injury more effectively than the monomer treatments. These findings identify UA‐18*β*GA NPs as a promising natural product‐based antiparasitic nanoformulation and support integrated screening as a practical strategy for discovering self‐assembling bioactive molecular combinations.

## Graphical abstract

An integrated computational screening strategy identified ursolic acid (UA) and 18*β*‐glycyrrhetinic acid (18*β*GA) as a self‐assembling food‐derived molecular pair. The resulting carrier‐free nanoparticles (UA‐18*β*GA) showed synergistic antiparasitic activity, reduced combined toxicity, and host‐protective anti‐inflammatory effects in zebrafish and murine disease models, highlighting a practical route for discovering self‐assembling bioactive nanomedicines.

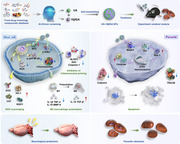

## 1 Introduction

Self‐assembled, carrier‐free nanodrugs have emerged as a promising strategy for therapeutic delivery because they offer high drug‐loading capacity, simple formulation, and the possibility of combining multiple bioactive molecules without introducing additional inert carriers [1, 2, 3]. In particular, self‐assembled nanoparticles composed of more than one active ingredient may improve therapeutic performance by integrating complementary pharmacological activities within a single nanosystem [4, 5, 6]. Despite these advantages, the identification of suitable molecular pairs for self‐assembly still relies heavily on empirical screening and trial‐ and‐error optimization [7]. Given the structural diversity of drug‐like molecules, this remains a major obstacle to the systematic development of carrier‐free nanomedicines. Therefore, computational strategies that can facilitate the identification of self‐assembling molecular combinations are of considerable interest.

Recent studies have begun to explore computational approaches for screening potential self‐assembling molecular pairs [8, 9]. Machine‐learning‐assisted methods based on two‐dimensional (2D) molecular fingerprints enable rapid virtual screening of large chemical libraries [8, 10, 11, 12], but 2D descriptors alone may not fully capture three‐dimensional (3D) spatial organization and stereoelectronic properties that are important for molecular recognition and noncovalent interactions during self‐assembly [13, 14, 15, 16, 17]. The incorporation of 3D molecular information may therefore provide complementary value, especially when shape matching and functional group geometry contribute to intermolecular association [18, 19, 20, 21, 22]. In parallel, the intermolecular basis of self‐assembly remains incompletely understood in many carrier‐free nanosystems [7, 23]. Experimental techniques such as FTIR, UV‐vis, fluorescence, and NMR spectroscopy can provide important information on molecular interactions after assembly [24, 25], whereas molecular dynamics (MD) simulations can further probe structural evolution at the molecular level [25, 26]. In this context, explainable artificial intelligence methods such as SHapley Additive exPlanations (SHAP) may serve as useful complementary tools for identifying molecular features associated with assembly‐related properties and for assisting model interpretation [27, 28, 29].

Malaria remains a major global health burden, and cerebral malaria (CM), one of the most severe complications of *Plasmodium falciparum* infection, is associated with substantial mortality and long‐term neurological sequelae [30]. Although antimalarial drugs remain indispensable, their effectiveness may be limited by resistance, toxicity, and treatment costs [31]. Bioactive food‐derived molecules represent an attractive source of candidate therapeutics because of their broad availability, relatively favorable safety profiles, and diverse pharmacological activities [32]. Using such molecules not only as therapeutic agents but also as building blocks for carrier‐free nanomedicines may provide an opportunity to combine intrinsic bioactivity with formulation advantages, including improved dispersibility and altered release behavior. Such an approach may be particularly relevant for parasitic diseases, in which both direct antiparasitic effects and host‐protective responses can influence therapeutic outcome.

**SCHEME 1:**
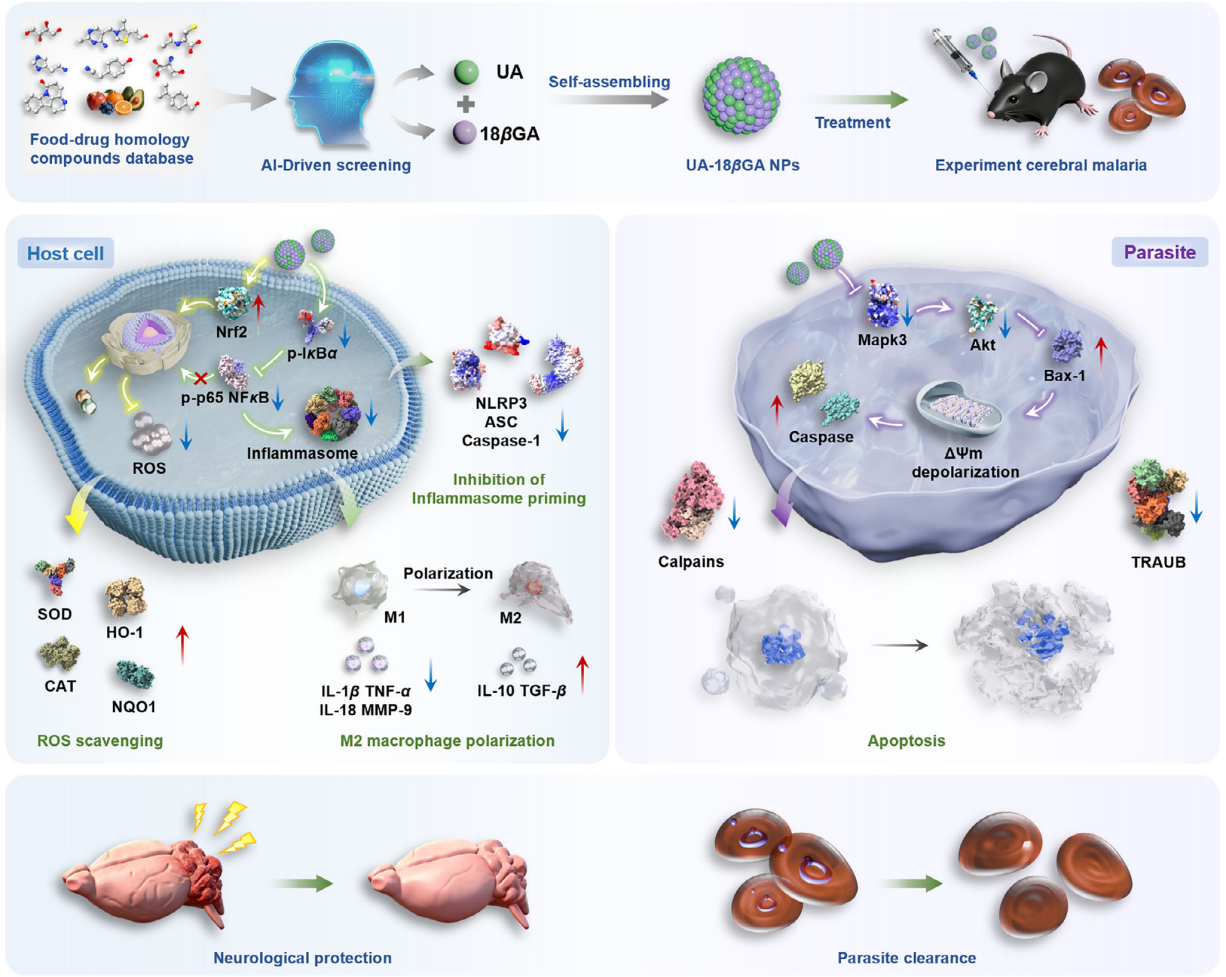
Computational screening identifies UA‐18*β*GA NPs with synergistic antiparasitic activity and host‐protective effects.

Here, we developed an integrated workflow combining computational screening and experimental validation to identify self‐assembling food‐derived molecular combinations with antiparasitic potential. This workflow combined 2D similarity screening, 3D pharmacophore matching, molecular docking, density functional theory (DFT) calculations, MD simulations, and exploratory SHAP‐assisted analysis. Applying this strategy to a food‐derived compound library led to the prioritization of ursolic acid (UA) and 18*β*‐glycyrrhetinic acid (18*β*GA), which were subsequently found to self‐assemble into stable nanoparticles (UA‐18*β*GA NPs). Their intermolecular interactions and structural evolution were investigated using combined spectroscopic characterization and computational analysis. The resulting nanoparticles exhibited synergistic antiparasitic activity in zebrafish parasite assays and showed therapeutic benefit in a murine model of experimental cerebral malaria, together with anti‐inflammatory and antioxidant effects (Scheme 1). These findings highlight UA‐18*β*GA NPs as a promising antiparasitic nanoformulation and support the use of integrated computational screening for the identification of self‐assembling bioactive molecular combinations.

## 2 Results

### 2.1 Integrated 2D/3D Screening Identifies UA and 18*β*GA as a Candidate Self‐Assembly Pair

**FIGURE 1:**
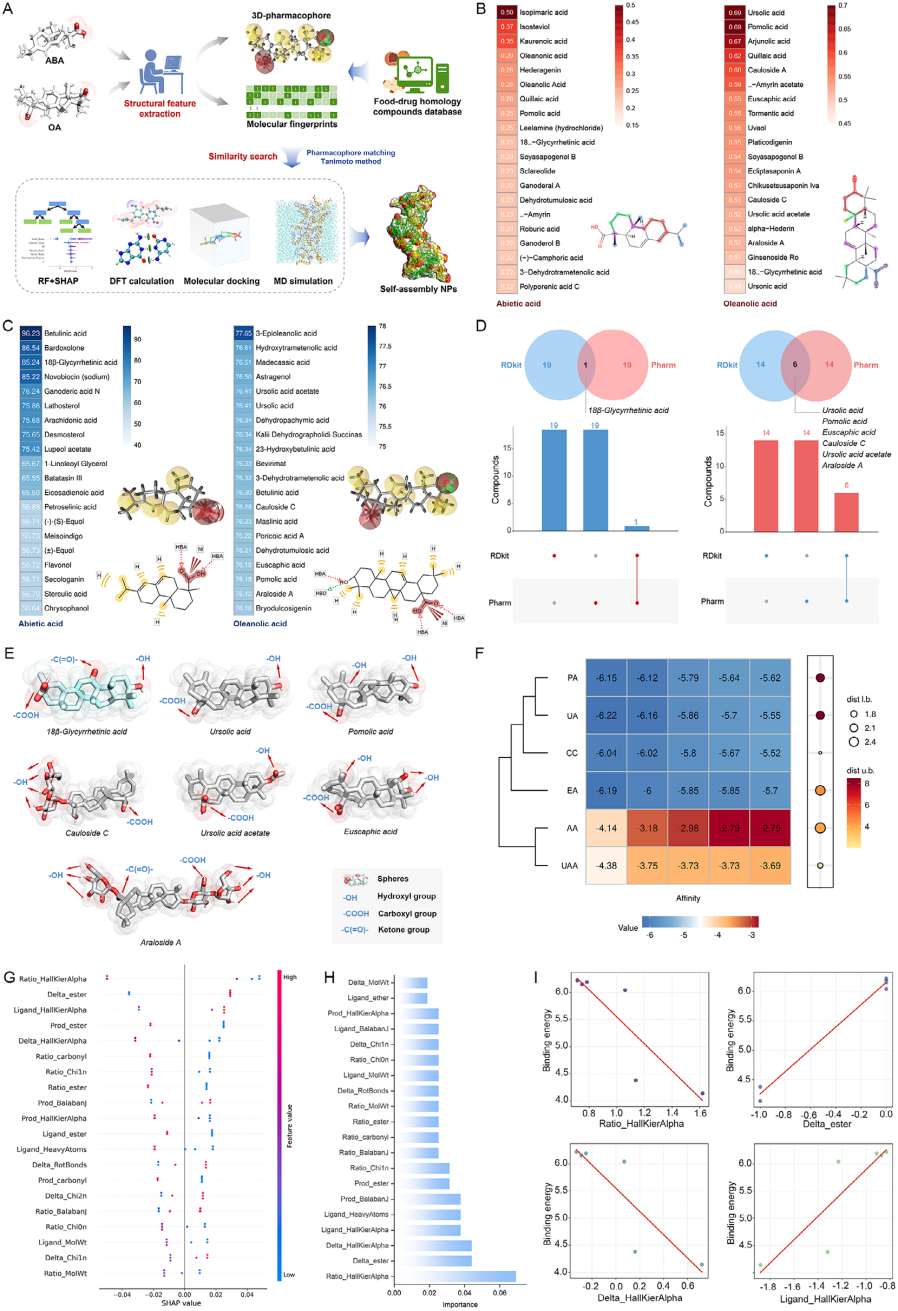
Integrated 2D/3D screening of UA‐18*β*GA self‐assembly. (A) Schematic illustration of the multi‐step virtual screening workflow integrating similarity‐based screening, pharmacophore matching, molecular docking, and exploratory feature analysis. (B) Top 20 candidate compounds ranked by structural similarity to ABA and OA based on RDKit fingerprint matching. (C) Top 20 candidate compounds identified by 3D pharmacophore matching using the chemical features of ABA and OA. (D) Overlap analysis of the similarity‐based and pharmacophore‐based screening results, showing the consensus candidate molecules. (E) 3D structures of the final candidate compounds, with functional groups potentially involved in intermolecular interactions highlighted in red. (F) Docking‐derived binding energies of representative molecular pair combinations among the selected candidates. (G) SHAP summary plot showing the top 20 molecular substructures contributing to the model output. (H) Random forest‐based feature importance ranking of molecular descriptors associated with binding energy prediction. (I) Correlation analysis between the top four fingerprint‐related features and docking‐derived binding energies.

To facilitate the identification of candidate self‐assembling molecular pairs, a multi‐step virtual screening workflow integrating structural and energetic criteria was established (Figure 1A). A food‐derived compound library was screened through a dual‐pathway strategy combining two‐dimensional (2D) molecular similarity analysis and three‐dimensional (3D) pharmacophore matching. Using RDKit‐based fingerprint similarity analysis, the top 20 candidate compounds showing structural similarity to the reference molecules abscisic acid (ABA) and oleanolic acid (OA) were identified (Figure 1B). Among these, isopimaric acid (similarity score, 0.50), isosteviol (0.37), and kaurenoic acid (0.35) showed relatively higher similarity to ABA, whereas ursolic acid (0.69), pomolic acid (0.69), and arjunolic acid (0.67) showed higher similarity to OA. In parallel, 3D pharmacophore matching based on the key chemical features of ABA and OA identified an additional set of top‐ranking candidates (Figure 1C). Representative compounds included betulinic acid (fit score, 96.23), bardoxolone (86.54), and 18*β*‐glycyrrhetinic acid (85.24) in the ABA‐matched set, as well as 3‐epioleanolic acid (77.65), hydroxytrametenolic acid (76.61), and madecassic acid (76.51) in the OA‐matched set. Comparison of the 2D and 3D screening results identified an overlapping set of candidate molecules (Figure 1D), including 18*β*‐glycyrrhetinic acid from the ABA‐related set and ursolic acid, pomolic acid, euscaphic acid, cauloside C, ursolic acid acetate, and araloside A from the OA‐related set. The 3D structures of these final candidates were then visualized to illustrate their spatial configurations and the distribution of functional groups potentially involved in intermolecular interactions (Figure 1E). To further assess intermolecular compatibility among the selected compounds, molecular docking was performed to estimate the binding affinities of potential self‐assembling pairs (Figure 1F; Figure S1). Among the tested combinations, the ursolic acid (UA)/18*β*‐glycyrrhetinic acid (18*β*GA) pair showed a relatively favorable predicted binding energy of −6.22 kcal/mol. On the basis of these screening and docking results, UA and 18*β*GA were prioritized for subsequent experimental validation.

To further explore structural features associated with intermolecular binding, an exploratory random forest (RF) analysis was performed, followed by SHapley Additive exPlanations (SHAP) analysis. The SHAP results highlighted the top 20 molecular substructures contributing to the model output (Figure 1G), whereas RF feature ranking identified descriptors that were relatively more important for binding energy prediction (Figure 1H). Correlation analysis further showed associations between docking‐derived binding energies and the top four fingerprint‐related features, namely Ratio_HallKierAlpha, Delta_ester, Delta_HallKierAlpha, and Ligand_HallKierAlpha (Figure 1I). These results indicate that spatial complementarity, molecular size matching, polarity/amphiphilicity balance, and hydrogen‐bond‐related features are important for favorable intermolecular interactions in this system. On this basis, UA and 18*β*GA were selected as a self‐assembling molecular pair for subsequent experimental validation.

### 2.2 Preparation and Characterization of UA‐18*β*GA NPs

**FIGURE 2:**
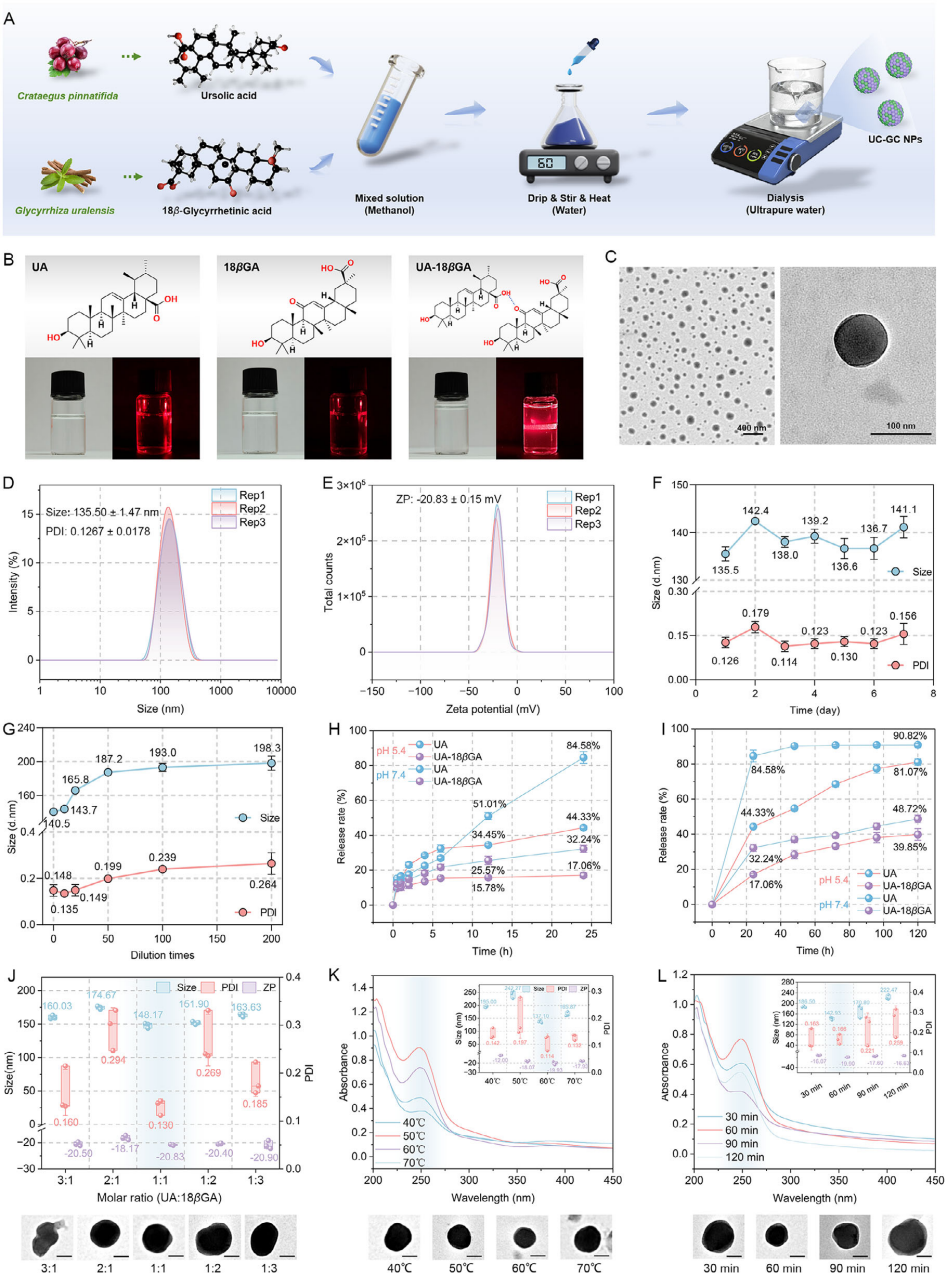
Preparation and characterization of UA‐18*β*GA NPs. (A) Schematic illustration of the self‐assembly process used to prepare UA‐18*β*GA NPs. (B) Tyndall effect observed in the aqueous dispersion of UA‐18*β*GA NPs. (C) Representative transmission electron microscopy (TEM) image of UA‐18*β*GA NPs. (D) Hydrodynamic diameter and polydispersity index (PDI) of UA‐18*β*GA NPs measured by dynamic light scattering (DLS). (E) Zeta potential of UA‐18*β*GA NPs. (F) Storage stability of UA‐18*β*GA NPs evaluated by monitoring hydrodynamic diameter and PDI during 7 days of storage in water at 4 °C. (G) Dilution stability of UA‐18*β*GA NPs evaluated by monitoring hydrodynamic diameter and PDI after 10‐ to 200‐fold dilution. In vitro release profiles of UA from free UA and UA‐18*β*GA NPs under physiological (pH 7.4) and acidic (pH 5.4) conditions over (H) 24 h and (I) 120 h. Optimization of the self‐assembly conditions based on TEM morphology, hydrodynamic diameter, PDI, zeta potential, and dilution stability as a function of (J) the initial UA/18*β*GA molar ratio, (K) assembly temperature, and (L) stirring duration. Scale bar: 100 nm.

Following the computational screening results, UA and 18*β*GA were selected for experimental self‐assembly, and the resulting nanoparticles were denoted as UA‐18*β*GA NPs. A schematic illustration of the preparation process is shown in Figure 2A. The formation of dispersed nanoparticles in water was initially indicated by the appearance of a clear Tyndall effect (Figure 2B; Figure S2). TEM imaging further showed that the prepared UA‐18*β*GA NPs exhibited a predominantly spherical morphology with a particle size of approximately 100 nm (Figure 2C). The physicochemical properties of UA‐18*β*GA NPs were further characterized by DLS. The nanoparticles showed an average hydrodynamic diameter of 135.5 ± 1.47 nm with a PDI of 0.1267 ± 0.0178, indicating a relatively narrow size distribution (Figure 2D). The zeta potential was measured to be −20.83 ± 0.15 mV (Figure 2E), consistent with the formation of a colloidally dispersed nanosystem. The stability of UA‐18*β*GA NPs was then evaluated under storage and dilution conditions. During storage in water at 4 °C for 7 days, the nanoparticles maintained hydrodynamic diameters in the range of 135.5–142.4 nm and PDI values between 0.114 and 0.179 (Figure 2F), indicating no obvious change in particle size distribution during the observation period. In the dilution stability assay, the nanoparticles remained dispersed after 10‐ and 200‐fold dilution, with hydrodynamic diameters ranging from 140.5 to 198.3 nm and PDI values ranging from 0.148 to 0.264 (Figure 2G). These data suggest that the nanoparticles retained their colloidal characteristics after substantial dilution. The release behavior of UA from UA‐18*β*GA NPs was evaluated under physiological (pH 7.4) and acidic (pH 5.4) conditions. As shown in Figure 2H, UA‐18*β*GA NPs showed slower release than free UA within 24 h under both pH 7.4 and pH 5.4 conditions. The cumulative release of free UA reached 84.58% and 44.33% at pH 7.4 and pH 5.4, respectively, whereas UA‐18*β*GA NPs released 32.24% and 15.95%. After extending the release period to 120 h, the cumulative release of free UA increased to 90.82% at pH 7.4 and 81.07% at pH 5.4, while the corresponding release values for UA‐18*β*GA NPs were 48.72% and 39.85%, respectively (Figure 2I). These results indicate that co‐assembly with 18*β*GA reduced the release rate of UA in both neutral and acidic media, and that release remained lower under acidic conditions in the present system.

To optimize the self‐assembly conditions, the effects of the initial UA/18*β*GA molar ratio, assembly temperature, and stirring duration were evaluated by TEM together with DLS analysis of particle size, PDI, zeta potential, and dilution stability (Figure 2J–L; Figure S3). When the UA/18*β*GA molar ratio was varied from 3:1 to 1:3, the particle morphology changed from irregular structures to more regular spherical nanoparticles and then to less uniform ellipsoidal particles as the proportion of 18*β*GA increased (Figure 2J). The 1:1 molar ratio produced the smallest and most uniform nanoparticles, with a mean hydrodynamic diameter of 148.17 nm, a PDI of 0.13, and a zeta potential of −20.83 mV. At higher 18*β*GA ratios, particle size increased and uniformity decreased. Using the 1:1 molar ratio, the assembly temperature was further optimized from 40 to 70 °C (Figure 2K). The absorbance increased with temperature and reached a maximum at 60 °C, which was accompanied by a more regular morphology, a reduced particle size of 137.1 nm, improved size uniformity, and maintained stability after 200‐fold dilution. Under the optimized molar ratio and temperature, the effect of stirring duration was then evaluated (Figure 2L). Compared with 30 min, assembly for 60 min yielded more regular nanoparticles with the smallest particle size, whereas prolonged stirring gradually increased particle size. In the 120 min group, the particles became more irregular, with the mean size increasing to 222.47 nm and the zeta potential decreasing to −16.63 mV. Based on these results, a UA/18*β*GA molar ratio of 1:1, an assembly temperature of 60 °C, and a stirring time of 60 min were selected for subsequent experiments.

### 2.3 Spectroscopic and Computational Analyses Reveal the Intermolecular Basis of UA‐18*β*GA Self‐Assembly

**FIGURE 3:**
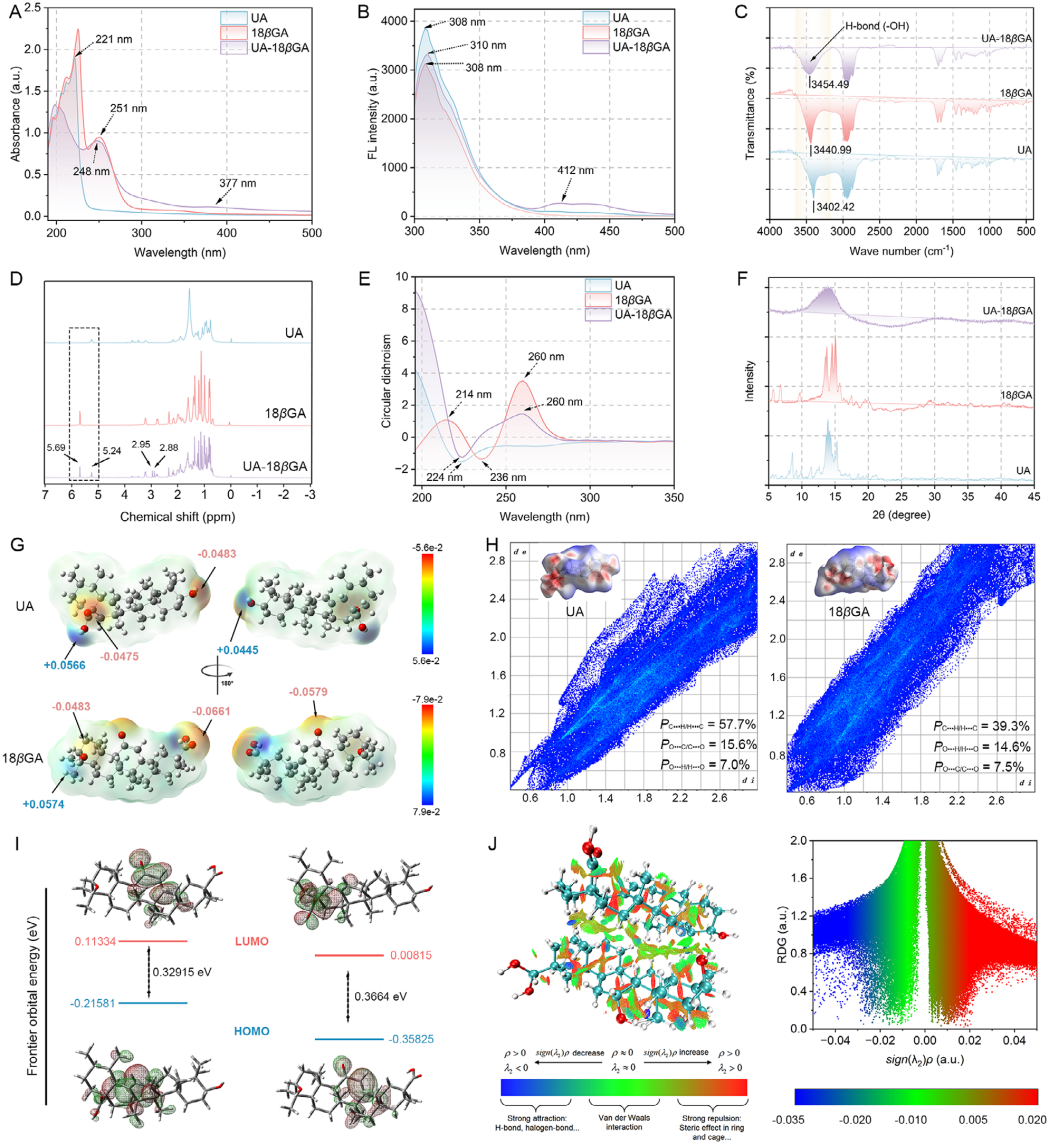
Mechanistic analysis of UA‐18*β*GA self‐assembly. (A) UV–vis absorption spectra of UA, 18*β*GA, and UA‐18*β*GA NPs. (B) Fluorescence emission spectra of UA, 18*β*GA, and UA‐18*β*GA NPs. (C) Fourier‐transform infrared (FT‐IR) spectra of UA, 18*β*GA, and UA‐18*β*GA NPs. (D) ^1^H NMR spectra of UA, 18*β*GA, and UA‐18*β*GA NPs in CDCl_3_. (E) Circular dichroism (CD) spectra of UA, 18*β*GA, and UA‐18*β*GA NPs. (F) X‐ray diffraction (XRD) patterns of UA, GA, and UA‐18*β*GA NPs. (G) Electrostatic potential (ESP) surfaces of UA and 18*β*GA. (H) Hirshfeld surfaces and corresponding two‐dimensional fingerprint plots of UA and 18*β*GA. (I) Calculated frontier molecular orbitals, including HOMO, LUMO, and the HOMO‐LUMO energy gap. (J) Non‐covalent interaction (NCl) analysis based on the reduced density gradient (RDG) method for the optimized UA/18*β*GA complex.

To investigate the intermolecular interactions associated with the self‐assembly of UA and 18*β*GA into UA‐18*β*GA NPs, combined spectroscopic and computational analyses were performed. In the UV–vis spectra, UA‐18*β*GA NPs retained characteristic absorption features of both components, while showing a slight blue shift from 251 to 248 nm together with the appearance of a new band at 377 nm (Figure 3A). These spectral changes suggested altered electronic interactions after self‐assembly. Consistently, fluorescence emission spectra showed a red shift from 308 to 310 nm and a new emission band at 412 nm in the nanoparticle formulation (Figure 3B), further supporting the formation of intermolecularly associated states in the self‐assembled system. Additional structural characterization provided further evidence for molecular reorganization after self‐assembly. FTIR spectra showed that the O–H stretching band of UA‐18*β*GA NPs shifted to 3454.49 cm^−^^1^ relative to those of the individual components (UA: 3402.42 cm^−^^1^; 18*β*GA: 3440.99 cm^−1^), accompanied by band broadening (Figure 3C), which was consistent with changes in hydrogen‐bonding environments. ^1^H NMR spectra preserved the characteristic proton signals of both UA and 18*β*GA, while also showing new resonances at 2.95 and 2.88 ppm (Figure 3D), suggesting altered local chemical environments in the self‐assembled state. Circular dichroism (CD) spectra further showed a marked change in the chiroptical response of UA‐18*β*GA NPs, including a negative band at 224 nm and a broad positive signal over 236–260 nm (Figure 3E), indicating supramolecular reorganization after self‐assembly. XRD analysis also showed broadened diffraction features in UA‐18*β*GA NPs relative to the monomeric compounds (Figure 3F), suggesting reduced crystallinity and a transition toward a more amorphous assembly state.

Computational analyses were then used to further examine the intermolecular interaction pattern between UA and 18*β*GA. Electrostatic potential (ESP) analysis showed charge complementarity between the carboxyl group of UA and the ester‐containing region of 18*β*GA (Figure 3G), supporting the possibility of favorable electrostatic interactions between the two molecules. Hirshfeld surface analysis and the corresponding 2D fingerprint plots indicated that both van der Waals contacts and hydrogen‐bond‐related interactions contributed to the intermolecular association, although their relative distributions differed between UA and 18*β*GA (Figure 3H). Frontier molecular orbital analysis further showed differences in the HOMO and LUMO energy levels of the two molecules (Figure 3I), suggesting the possibility of charge redistribution upon molecular association. In addition, non‐covalent interaction (NCI) analysis based on the reduced density gradient showed extensive green isosurfaces distributed across the UA/18*β*GA complex, together with localized regions consistent with hydrogen‐bonding interactions (Figure 3J), indicating that van der Waals interactions, along with hydrogen bonding, may contribute to stabilization of the self‐assembled structure. These results indicate that the UA/18*β*GA self‐assembled structure is stabilized by van der Waals interactions, together with electrostatic complementarity and hydrogen bonding.

### 2.4 Molecular Dynamics Analysis of UA‐18*β*GA Self‐Assembly

**FIGURE 4:**
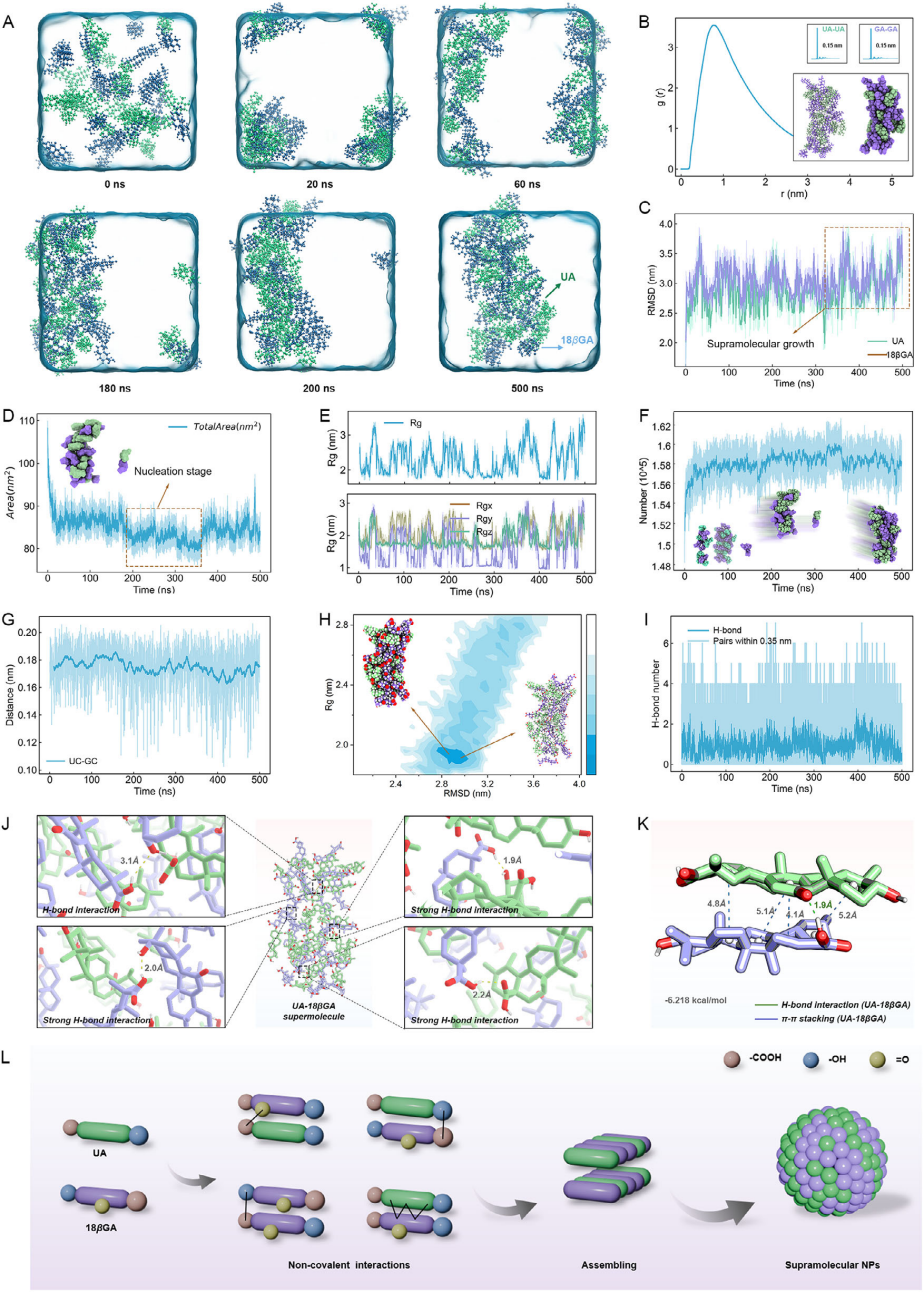
Molecular dynamics analysis of UA‐18*β*GA self‐assembly. (A) Representative snapshots from the 500 ns MD simulation trajectory showing the time‐dependent self‐assembly process of UA (green) and 18*β*GA (purple) in water. (B) Radial distribution function (RDF) between UA and 18*β*GA during the simulation. Time evolution of key structural parameters during the simulation, including (C) root‐mean‐square deviation (RMSD), (D) solvent‐accessible surface area (SASA), and (E) radius of gyration (*R*_g_). (F, G) Time‐dependent intermolecular contact analysis of the UA and 18*β*GA system, including contact frequency and mean intermolecular distance. (H) Free energy landscape projected onto the *R*_g_ and RMSD coordinates, with representative structures corresponding to major energy basins. (I) Time evolution of the number of hydrogen bonds formed during the simulation. (J) Representative intermolecular interaction patterns in the UA‐18*β*GA assembled structure. (K) Molecular docking prediction of a representative binding mode between UA and 18*β*GA. (L) Schematic model summarizing the proposed self‐assembly process of UA and 18*β*GA.

To further examine the dynamic evolution of UA‐18*β*GA self‐assembly, molecular dynamics (MD) simulations were performed over 500 ns. The simulation trajectory showed progressive organization of initially dispersed UA and 18*β*GA molecules into a more compact aggregate during the simulation period (Figure 4A). The radial distribution function displayed a pronounced peak at approximately 0.8 nm with a *g*(*r*) value of about 3.5 (Figure 4B), suggesting a preferred intermolecular distance between the two components during assembly. The time evolution of several structural parameters further supported the formation of a relatively stable assembled state. The root‐mean‐square deviation (RMSD) gradually approached a plateau after approximately 200 ns (Figure 4C), indicating reduced structural fluctuation at later stages of the simulation. At the same time, the solvent‐accessible surface area (SASA) decreased from about 110 to 82 nm^2^ (Figure 4D), consistent with reduced solvent exposure during assembly. The radius of gyration (*R*_g_) also decreased over time (Figure 4E), suggesting progressive structural compaction of the molecular aggregate. Intermolecular contact analysis showed that the contact frequency increased from 148 000 to 158 000, whereas the mean intermolecular distance decreased from 0.182 to 0.163 nm during the simulation (Figure 4F,G). These changes were consistent with progressively closer intermolecular association. In addition, the free energy landscape projected onto the *R*_g_ and RMSD coordinates showed a major low‐energy basin (Figure 4H), indicating the presence of a relatively stable conformational state in the assembled system. Hydrogen‐bond analysis further showed that, after the initial association stage, the number of intermolecular hydrogen bonds remained at approximately six for most of the simulation period (Figure 4I). Representative interaction patterns suggested hydrogen‐bond formation between the carboxyl group of UA and the carboxyl or hydroxyl groups of 18*β*GA, together with intramolecular hydrogen bonds within 18*β*GA (Figure 4J). Molecular docking yielded a predicted binding affinity of ‐6.22 kcal/mol and suggested a binding mode involving hydrogen bonding between the carboxyl group of UA and the ketone group of 18*β*GA, together with extensive hydrophobic contacts between their rigid cyclic scaffolds (Figure 4K). The MD simulation and docking results support a multistep self‐assembly process for UA and 18*β*GA, beginning with intermolecular recognition and followed by structural compaction and stabilization through hydrogen bonding, van der Waals interactions, and hydrophobic association, resulting in a stable co‐assembled structure (Figure 4L).

### 2.5 Enhanced Biosafety, Cellular Uptake, and In Vivo Distribution of UA‐18*β*GA NPs in Zebrafish Models

**FIGURE 5:**
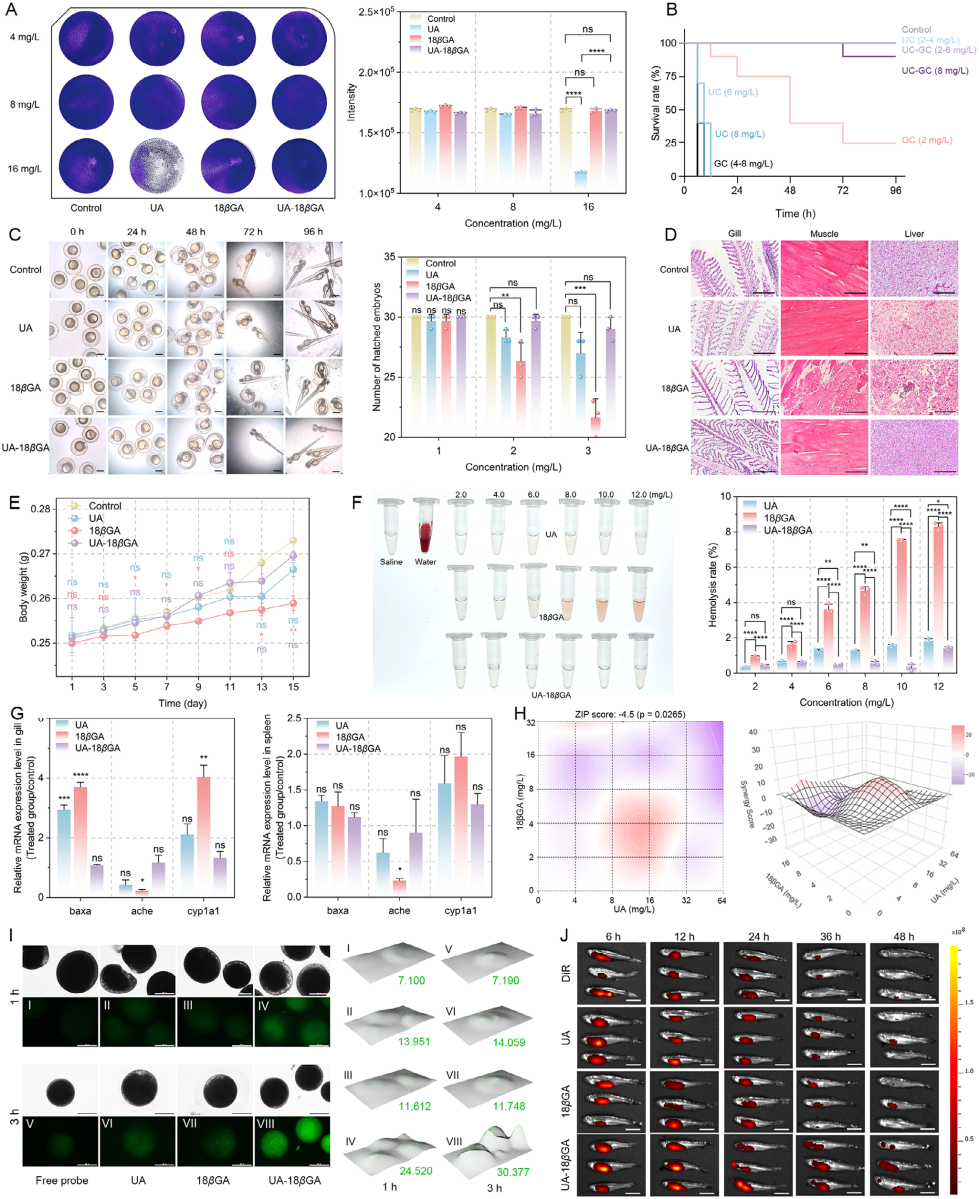
Biosafety, cellular uptake, and in vivo distribution of UA‐18*β*GA NPs in zebrafish models. (A) Viability of EPC cells after 48 h exposure to UA, 18*β*GA, and UA‐18*β*GA NPs at concentrations of 4, 8, and 16 mg/L. (B) Survival rates of zebrafish exposed to UA, 18*β*GA, and UA‐18*β*GA NPs at concentrations ranging from 2 to 8 mg/L for 96 h (*n* = 20). (C) Developmental phenotypes of zebrafish embryos (*n* = 30) after 96 h treatment with UA, 18*β*GA, and UA‐18*β*GA NPs at 1–3 mg/L. Scale bar: 300 µm. (D) H&E‐stained sections of gill, muscle, and liver tissues from zebrafish after 96 h exposure to 3 mg/L UA, 18*β*GA, or UA‐18*β*GA NPs. Scale bar: 100 µm. (E) Body weight changes of zebrafish after 15 days of daily oral administration of UA, 18*β*GA, or UA‐18*β*GA NPs. (F) Hemolysis assay of UA, 18*β*GA, and UA‐18*β*GA NPs. (G) Relative expression levels of toxicity‐related genes in the gill and liver of zebrafish after exposure to UA, 18*β*GA, or UA‐18*β*GA NPs (2 mg/L for 24 h) (*n* = 3). (H) ZIP model‐based analysis of the combined cytotoxic effects of UA and 18*β*GA on cell viability. (I) Fluorescence images showing uptake of FITC‐labeled UA, 18*β*GA, and UA‐18*β*GA NPs (0.5 mg/L) by *I. multifiliis* (*n* = 30) after 1 and 3 h incubation. Scale bar: 200 µm. (J) in vivo fluorescence imaging of zebrafish after administration of free DiR, DiR‐labeled UA, DiR‐labeled 18*β*GA, or DiR‐labeled UA‐18*β*GA NPs (*n* = 3). Scale bar: 1 cm. Data are presented as the mean ± SD. An unpaired 2‐tailed *t*‐test was used in (G). Statistical significance in panels (A, C, E, and F) was analyzed using one‐way ANOVA followed by Tukey's multiple comparisons test. ****p < 0.0001, ***p < 0.001, **p < 0.01, *p < 0.05, ^ns^ *p* > 0.05.

A comprehensive biosafety evaluation of UA‐18*β*GA NPs was performed using both in vitro and in vivo zebrafish‐related models. After 48 h exposure, EPC cell viability remained above 95% in the UA‐18*β*GA NP group at 16 mg/L, whereas UA alone reduced cell viability to approximately 20% at the same concentration (Figure 5A). These results indicated that self‐assembly markedly reduced the cytotoxicity of the free components. The acute toxicity of the formulation was further evaluated in zebrafish. UA caused rapid lethality at concentrations above 6 mg/L, whereas 18*β*GA treatment also induced marked mortality over the 96 h observation period. In contrast, zebrafish exposed to 6 mg/L UA‐18*β*GA NPs maintained 100% survival throughout the test period without obvious signs of toxicity (Figure 5B). In the embryotoxicity assay, 18*β*GA significantly impaired embryonic development, whereas the UA‐18*β*GA NP group showed a markedly higher hatching rate and remained comparable to the control group under the tested conditions (Figure 5C). Histopathological examination further showed that zebrafish treated with free UA or 18*β*GA exhibited tissue injury, including gill filament contraction and hepatocyte shrinkage, whereas the UA‐18*β*GA NP‐treated group retained relatively intact tissue architecture (Figure 5D). Consistently, during 15 days of oral administration, 18*β*GA‐treated zebrafish showed reduced body weight gain, whereas zebrafish treated with UA‐18*β*GA NPs displayed growth patterns comparable to those of the control group (Figure 5E). The improved biocompatibility of the nanoformulation was also supported by the hemolysis assay. Both UA and 18*β*GA caused concentration‐dependent hemolysis, whereas UA‐18*β*GA NPs showed markedly lower hemolytic activity across the tested concentration range (Figure 5F). In addition, toxicity‐related gene expression analysis in gill and liver tissues showed that free UA and 18*β*GA significantly altered the expression of *baxa*, *ache*, and *cyp1a1*, whereas expression levels in the UA‐18*β*GA NP group remained closer to baseline (Figure 5G). The changes were more pronounced in gill tissue, suggesting greater tissue sensitivity to the free compounds. After evaluating the cytotoxicity, hemolysis, embryotoxicity, and acute toxicity of UA, 18*β*GA, and UA‐18*β*GA NPs, ZIP analysis was further used to assess the combined effects of UA and 18*β*GA on cell viability. The combination showed a negative ZIP score (−4.5, *p* = 0.0265), indicating an antagonistic interaction in cytotoxicity and supporting a reduced combined toxic effect after co‐assembly (Figure 5H).

To determine whether the nanoparticles retained efficient biological accessibility, uptake and distribution studies were performed. Fluorescence imaging showed that FITC‐labeled UA‐18*β*GA NPs were internalized by *I. multifiliis* more efficiently than the free probe, free UA, or free 18*β*GA (Figure 5I). This uptake preference became more pronounced over time, and intracellular fluorescence at 3 h was markedly stronger than that at 1 h. in vivo fluorescence imaging further showed that DiR‐labeled UA‐18*β*GA NPs remained widely distributed in zebrafish up to 48 h after administration and gradually accumulated in internal organs and gill tissues during circulation (Figure 5J; Figure S4). By contrast, the free‐drug groups showed weaker fluorescence signals, and no obvious fluorescence was detected in the gills. These findings indicate that UA‐18*β*GA NPs possess enhanced parasite uptake and prolonged in vivo retention with broader tissue distribution.

### 2.6 UA‐18*β*GA NPs Exhibit Synergistic Antiparasitic Activity and Induce Parasite Apoptosis

**FIGURE 6:**
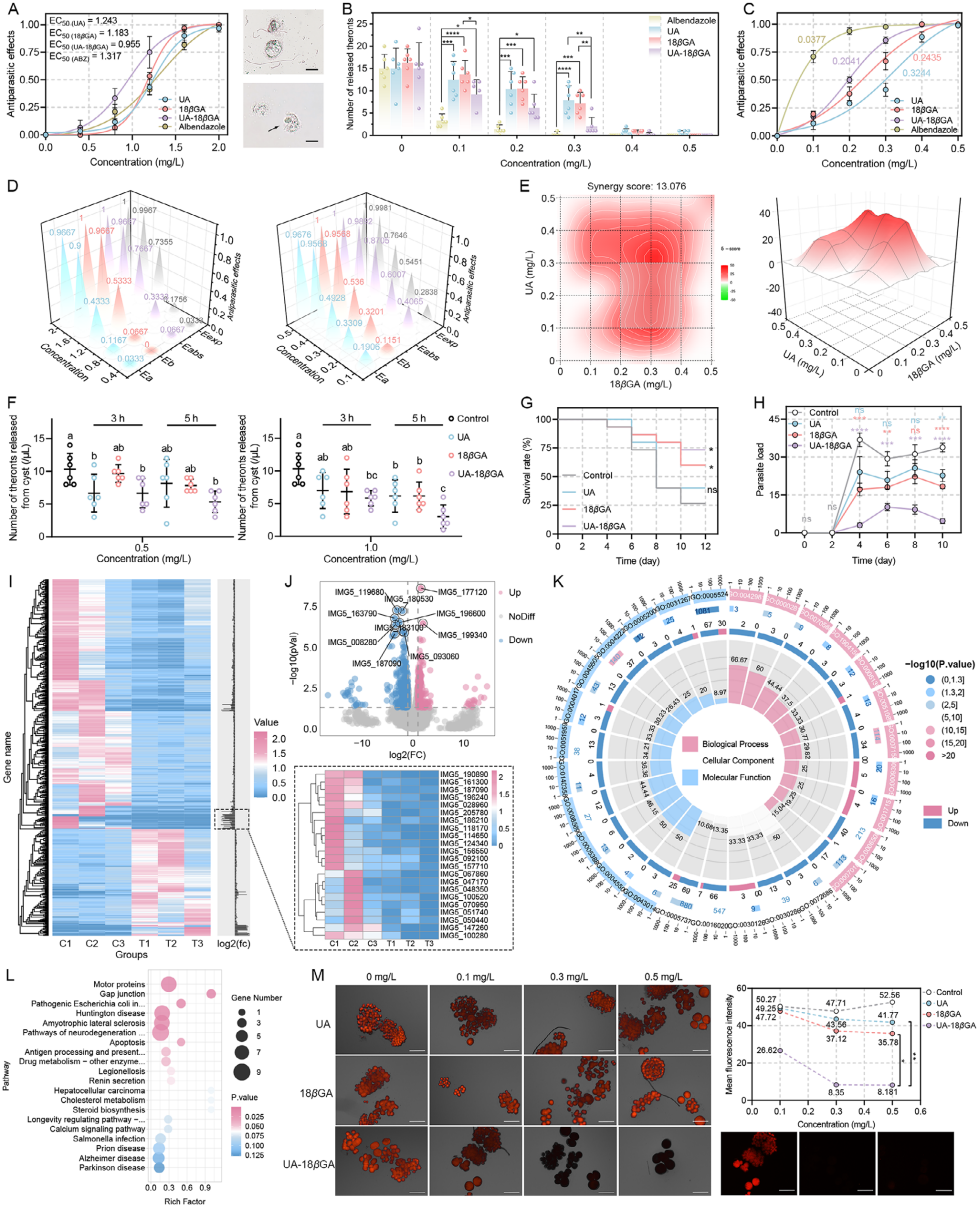
Synergistic antiparasitic activity of UA‐18*β*GA NPs in zebrafish. (A) In vitro antiparasitic activity of UA‐18*β*GA NPs, UA, and 18*β*GA against *I. multifiliis* theronts (*n* = 300). Albendazole (ABZ) was used as the positive control. Insets show representative microscopic images of parasite death after treatment with UA‐18*β*GA NPs. Scale bar: 50 µm. (B) Inhibitory effects of UA‐18*β*GA NPs on parasite proliferation. (C) Dose‐response inhibition curves and half‐maximal effective concentration (EC_50_) values of UA, 18*β*GA, and UA‐18*β*GA NPs against parasite cysts (*n* = 30). (D) Bliss model‐based analysis of the activities of UA alone (*E*_a_), 18*β*GA alone (*E*_b_), the observed combined activity (*E*_abs_), and the expected combined activity (*E*_exp_) at the theront and tomont stages. (E) Highest single agent (HSA)‐based synergy score maps and corresponding three‐dimensional response surface plots for the combined antiparasitic activity of UA and 18*β*GA. (F) in vivo antiparasitic efficacy after treatment with UA, 18*β*GA, or UA‐18*β*GA NPs at 0.5 and 1.0 mg/L (*n* = 30). (G) Survival rates and (H) parasite burdens in infected fish after oral administration of UA, 18*β*GA, and UA‐18*β*GA NPs at 50 mg/kg. (I) Heatmap of differentially expressed genes between the control group (C1, C2, and C3) and the UA‐18*β*GA NP‐treated group (T1, T2, and T3). (J) Volcano plot showing significantly upregulated and downregulated genes. (K) Circular plot of Gene Ontology (GO) enrichment terms associated with the differentially expressed genes. (L) Top 20 enriched KEGG pathways identified in the treated group. (M) Fluorescence microscopy images showing cell‐cycle arrest in parasites treated with UA‐18*β*GA NPs, compared with those treated with UA or 18*β*GA alone (*n* = 30). Scale bar: 300 µm. Data are presented as the mean ± SD. Statistical significance in panels (B, F, H, and M) was analyzed using one‐way ANOVA followed by Tukey's multiple comparisons test. ****p < 0.0001, ***p < 0.001, **p < 0.01, *p < 0.05, ^ns^ *p* > 0.05.

The antiparasitic activity of UA‐18*β*GA NPs against the protozoan parasite *I. multifiliis* was evaluated at multiple developmental stages. In vitro assays showed that UA‐18*β*GA NPs displayed stronger antiparasitic activity than either UA or 18*β*GA alone, with an EC_50_ of 0.955 mg/L, compared with 1.243 mg/L for UA and 1.183 mg/L for 18*β*GA (Figure 6A). Representative microscopic images showed marked parasite death and membrane disruption after treatment with UA‐18*β*GA NPs. The nanoparticles also suppressed parasite proliferation (Figure 6B) and showed increased activity against parasite cysts, with an EC_50_ of 0.2041 mg/L, compared with 0.3244 mg/L for UA and 0.2435 mg/L for 18*β*GA (Figure 6C). In addition, microscopic examination showed that UA‐18*β*GA NPs at 0.1–0.5 mg/L more effectively disrupted parasite reproductive stages than the individual compounds, with a higher proportion of dead and undifferentiated parasites observed in the nanoparticle‐treated groups (Figure S5). To quantitatively assess whether the enhanced antiparasitic activity reflected true synergy rather than simple additivity, combination effects were analyzed using Bliss independence and highest single agent (HSA) reference models. Bliss analysis showed that the observed combined activity of UA and 18*β*GA exceeded the expected additive activity across the tested concentrations at both the theront and tomont stages (Figure 6D). Consistently, HSA analysis yielded a positive synergy score of 13.076 (Figure 6E), supporting a synergistic interaction between UA and 18*β*GA in the antiparasitic assays. The in vivo therapeutic activity of UA‐18*β*GA NPs was further evaluated by bath immersion and oral administration. Bath treatment with UA‐18*β*GA NPs (0.5 and 1.0 mg/L for 3 and 5 h) effectively reduced trophont burden in situ and showed stronger antiparasitic activity than the corresponding free compounds (Figure 6F). After oral administration, UA‐18*β*GA NPs increased the survival rate of infected fish to 75%, compared with 25% in the infected control group, 40% in the UA group, and 60% in the 18*β*GA group (Figure 6G). In parallel, parasite burden was reduced to approximately 10 in the UA‐18*β*GA NP‐treated group, compared with approximately 20 and 16 in the UA‐ and 18*β*GA‐treated groups, respectively (Figure 6H). These results show that self‐assembly of UA and 18*β*GA enhanced antiparasitic efficacy in both in vitro and in vivo models.

To investigate the biological responses associated with the antiparasitic activity of UA‐18*β*GA NPs, transcriptomic analysis was performed on parasites after treatment (Figure S6). Principal component analysis showed clear separation between the untreated group (C1, C2, and C3) and the UA‐18*β*GA NP‐treated group (T1, T2, and T3), indicating substantial transcriptomic differences after treatment (Figure S7). Hierarchical clustering revealed that a large proportion of differentially expressed genes were downregulated in the treated parasites (Figure 6I), including genes related to ion transport (IMG5_114650), dynein motor (IMG5_048350), and kelch motif‐containing proteins (IMG5_147260). Volcano plot analysis identified 460 downregulated genes and 196 upregulated genes (Figure 6J). Functional enrichment analysis further suggested that the differentially expressed genes were associated with biological processes and pathways relevant to parasite survival and cellular homeostasis (Figure 6K). Gene Ontology analysis showed enrichment in processes including cell adhesion, proteolysis, and calcium ion transport, as well as functions related to the dynein complex, mitotic spindle, replication vesicle, metal endonuclease activity, microtubule binding, and microtubule motor activity (Figure S8). KEGG pathway analysis identified motor protein, gap junction, and apoptosis among the top enriched pathways (Figure 6L). To further examine the cellular effects of UA‐18*β*GA NPs, cell‐cycle and apoptosis‐related fluorescence assays were performed. Parasites treated with UA‐18*β*GA NPs showed markedly reduced fluorescence signals compared with those treated with UA or 18*β*GA alone, particularly at 0.3 and 0.5 mg/L, where fluorescence was nearly absent (Figure 6M). These findings, together with the transcriptomic results, support that UA‐18*β*GA NPs were associated with cell‐cycle disruption and apoptosis‐related responses in the parasites.

### 2.7 UA‐18*β*GA NPs Induce Parasite Apoptosis Through Erk1/Akt‐Associated Signaling

**FIGURE 7:**
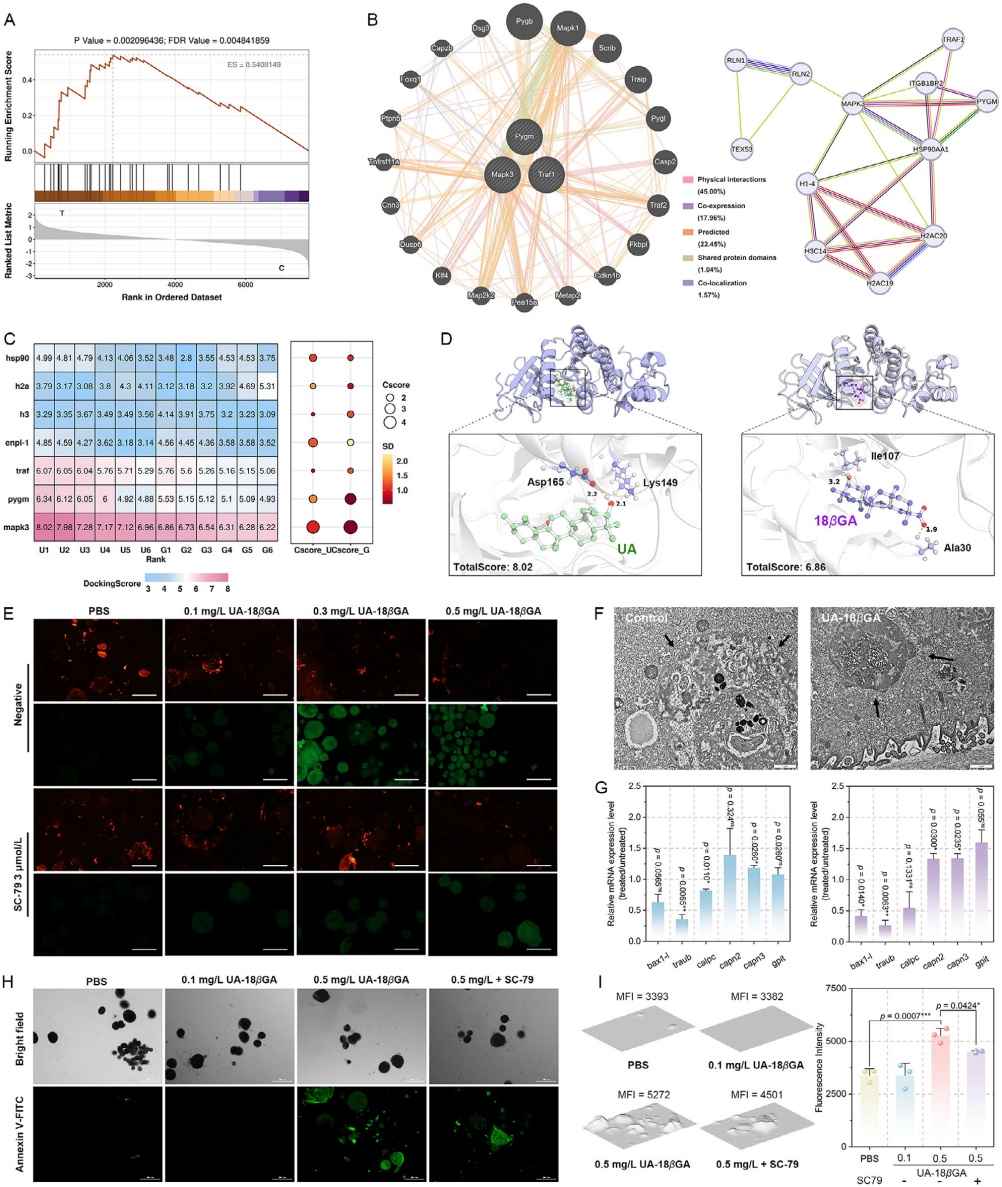
Apoptotic mechanism of UA‐18*β*GA NPs in parasites. (A) Gene set enrichment analysis (GSEA) of apoptosis‐related gene signatures in parasites after UA‐18*β*GA NP treatment. (B) Protein–protein interaction (PPI) network of apoptosis‐related differentially expressed genes (DEGs), constructed using the GeneMANIA and STRING databases. (C) Docking‐derived binding energies between key PPI‐network proteins and UA or 18*β*GA, obtained by semi‐flexible molecular docking. (D) Predicted binding poses of UA (green) and 18*β*GA (purple) in the active site of Mapk3. (E) Assessment of mitochondrial membrane potential (ΔΨ_m_) in parasites treated with UA‐18*β*GA NPs in the absence or presence of the Akt activator SC‐79. Scale bar: 300 µm. (F) TEM images of parasites after UA‐18*β*GA NP treatment. The arrow indicates the parasite nucleus. Scale bar: 1 µm. (G) Relative expression levels of apoptosis‐related genes in parasites after treatment with UA‐18*β*GA NPs (*n* = 3). (H) Annexin V‐FITC staining of parasites after treatment with UA‐18*β*GA NPs in the absence or presence of SC‐79. Scale bar: 300 µm. (I) Quantification of the mean fluorescence intensity (MFI) of Annexin V‐FITC staining from panel H. Data are presented as the mean ± SD. An unpaired 2‐tailed *t*‐test was used in (G). Statistical significance in (I) was analyzed using one‐way ANOVA followed by Tukey's multiple comparisons test. ***p < 0.001, **p < 0.01, *p < 0.05, ^ns^ *p* > 0.05.

Based on the transcriptomic evidence indicating apoptosis‐related responses, we further examined signaling pathways potentially involved in UA‐18*β*GA NP‐induced parasite death. Gene set enrichment analysis (GSEA) showed significant changes in apoptosis‐related gene signatures after treatment (Figure 7A). Protein‐protein interaction (PPI) network analysis of apoptosis‐related differentially expressed genes, performed using the STRING and GeneMANIA databases, identified several highly connected nodes, including *pygm*, *mapk3*, and *traf1* (Figure 7B), suggesting that these factors may participate in the observed death‐related response. To further explore potential molecular targets, seven candidate hub proteins were subjected to semi‐flexible molecular docking with UA and 18*β*GA. Among them, Mapk3 showed the most favorable predicted interactions, with docking scores of 8.02 for UA and 6.86 for 18*β*GA (Figure 7C; Figure S9). The predicted binding poses suggested that UA formed hydrogen bonds with Asp165 (2.2 Å) and Lys149 (2.1 Å), whereas 18*β*GA formed hydrogen bonds with Ile107 (3.2 Å) and Ala30 (1.9 Å) within the Mapk3 binding pocket (Figure 7D). Additional molecular dynamics simulations showed stable interaction patterns for these complexes over the simulated trajectory (Figure S10), supporting the possibility that Mapk3 may be involved in the biological response to UA‐18*β*GA NP treatment.

Functional assays further showed that UA‐18*β*GA NP treatment reduced the mitochondrial membrane potential (ΔΨ_m_), and this effect was partially reversed by co‐treatment with the Akt activator SC‐79 (Figure 7E). TEM imaging also showed ultrastructural alterations in treated parasites, including nuclear condensation and structural disruption (Figure 7F). In parallel, qPCR analysis showed concentration‐dependent changes in apoptosis‐related gene expression after UA‐18*β*GA treatment, including downregulation of *bax1‐i*, *traub*, and *calpc*, together with upregulation of *capn2*, *capn3*, and *gpit* (Figure 7G). The involvement of Akt‐related signaling was further supported by rescue experiments. Annexin V‐FITC staining showed that co‐treatment with SC‐79 attenuated UA‐18*β*GA NP‐induced apoptotic signals (Figure 7H; Figure S11), and quantitative analysis of the mean fluorescence intensity showed an approximately 30% reduction compared with UA‐18*β*GA NP treatment alone (Figure 7I). These results indicate that UA‐18*β*GA NPs induce parasite apoptosis and that this effect involves modulation of the Erk1/Akt signaling axis.

### 2.8 Antioxidant and Anti‐Inflammatory Effects of UA‐18*β*GA NPs in Zebrafish

**FIGURE 8:**
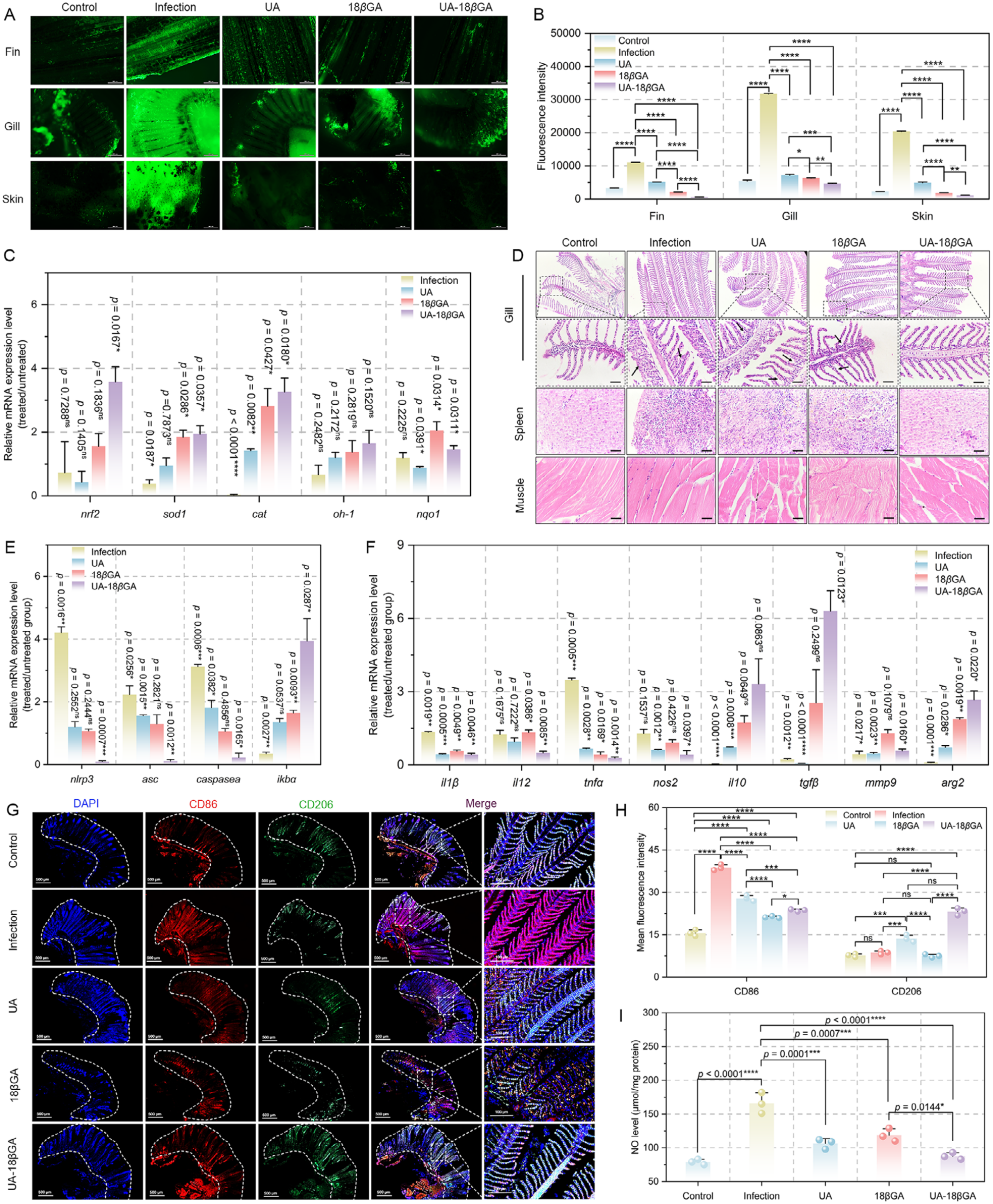
Antioxidant and anti‐inflammatory effects of UA‐18*β*GA NPs in zebrafish. (A) Detection of intracellular reactive oxygen species (ROS) in fin, gill, and skin tissues from the control, infected, UA‐, 18b*β*GA‐, and UA‐18*β*GA NP‐treated groups (1.0 mg/L, 6 h). Scale bar: 300 µm. (B) Quantification of DCFH‐DA mean fluorescence intensity (MFI) from panel A. (C) Relative mRNA expression levels of antioxidant‐response‐related genes (*n* = 3). (D) H&E‐stained sections of gill, spleen, and muscle tissues from the indicated groups (1.0 mg/L, 6 h). Scale bar: 10 µm. (E) Relative expression levels of genes associated with inflammasome priming (*n* = 3). (F) Relative expression levels of genes associated with macrophage polarization and inflammatory regulation (*n* = 3). (G) Immunofluorescence staining of M1 (CD86, red) and M2 (CD206, green) macrophage markers in zebrafish gill tissue, with nuclei counterstained with DAPI (blue). Scale bar: 500 µm. (H) Quantification of fluorescence intensity from panel G. (I) Nitric oxide levels in zebrafish tissues from the indicated treatment groups (1.0 mg/L, 6 h) (*n* = 3). Data are presented as the mean ± SD. An unpaired 2‐tailed *t*‐test was used in panels (C–F). Statistical significance in panels (B, H, and I) was analyzed using one‐way ANOVA followed by Tukey's multiple comparisons test. ****p < 0.0001, ***p < 0.001, **p < 0.01, *p < 0.05, ^ns^ *p* > 0.05.

Because parasitic infection is accompanied by oxidative stress and inflammatory injury in host tissues, the antioxidant and anti‐inflammatory effects of UA‐18*β*GA NPs were further evaluated in infected zebrafish. Treatment with UA‐18*β*GA NPs (1.0 mg/L, 6 h) reduced infection‐associated reactive oxygen species (ROS) signals in fin, gill, and skin tissues, as indicated by lower DCFH‐DA fluorescence intensity relative to the infected group and the corresponding monomer‐treated groups (Figure 8A,B). Consistent with this observation, qPCR analysis showed increased expression of antioxidant‐response‐related genes, including *nrf2*, *ho‐1*, *nqo1*, *sod1*, and *cat*, in the UA‐18*β*GA NP‐treated group (Figure 8C). Histological examination further showed that UA‐18*β*GA NP treatment alleviated infection‐associated tissue injury in gill, spleen, and muscle tissues compared with the infected group (Figure 8D). To further examine inflammation‐related responses, the expression of genes associated with inflammasome priming was analyzed. UA‐18*β*GA NP treatment reduced the mRNA levels of *nlrp3*, *asc*, and *caspase‐a*, while increasing the expression of *ikbβ* (Figure 8E), suggesting modulation of inflammation‐related signaling during treatment.

Changes in macrophage‐associated and inflammatory‐response genes were also observed after UA‐18*β*GA NP treatment. Compared with the infected group, the nanoparticle‐treated group showed lower expression of pro‐inflammatory genes, including *il1β*, *il12*, *tnfα*, and *nos2a*, together with higher expression of anti‐inflammatory mediators such as *il10* and *tgfβ* (Figure 8F). In addition, genes associated with M2‐like macrophage responses, including *arg2* and *mmp9*, were increased. Immunofluorescence staining of zebrafish gill tissue further showed reduced CD86‐associated signals and increased CD206‐associated signals after UA‐18*β*GA NP treatment (Figure 8G), and quantitative image analysis supported this shift in marker expression (Figure 8H). Consistent with these results, nitric oxide levels were also decreased in the UA‐18*β*GA NP‐treated group (Figure 8I). Taken together, these results support that UA‐18*β*GA NPs reduced oxidative stress and were associated with anti‐inflammatory host responses in infected zebrafish, including altered macrophage marker expression, lower nitric oxide levels, and reduced expression of inflammasome‐ and pro‐inflammatory‐response‐related genes.

### 2.9 UA‐18*β*GA NPs Improve Therapeutic Outcomes and Suppress Pathogenic Inflammation in Experimental Cerebral Malaria

**FIGURE 9:**
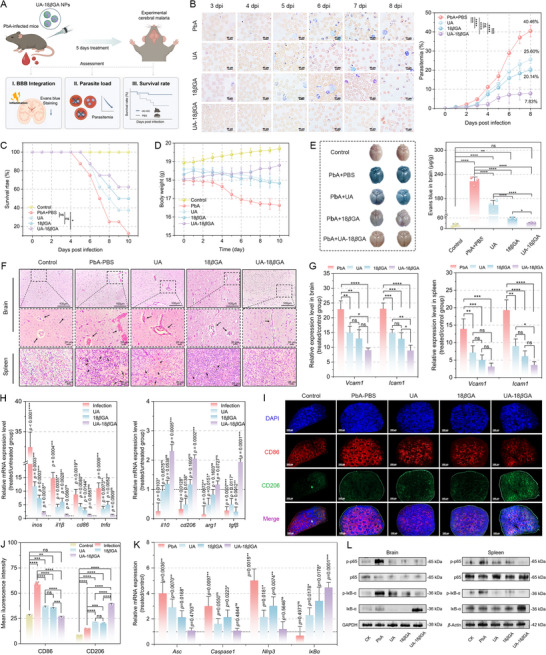
Therapeutic efficacy and mechanism of UA‐18*β*GA NPs in experimental cerebral malaria. (A) Schematic of the in vivo treatment regimen. (B) Left: Representative microscopic images of infected red blood cells (iRBCs) in the control, UA‐, 18*β*GA‐, and UA‐18*β*GA NP‐treated groups. Right: Parasitemia progression over 8 days post‐infection (dpi). Scale bar: 10 µm. (C) Body weight changes in uninfected, PbA‐infected, UA‐treated, 18*β*GA‐treated, and UA‐18*β*GA NP‐treated mice (50 mg/kg) during the 8‐day observation period. (D) Survival curves of the indicated treatment groups over 8 dpi (*n* = 8). (E) Blood‐brain barrier (BBB) integrity assessment by Evans blue extravasation. Left: Representative brain images. Right: Quantification of Evans blue leakage. (F) H&E‐stained sections of brain and spleen tissues from the indicated groups. Scale bar: 20 µm. (G) Relative mRNA expression levels of the adhesion molecules *Vcam‐1* and *Icam‐1* in brain and spleen tissues at 8 dpi. (H) Relative mRNA expression levels of inflammation‐related genes in spleen tissues from the indicated groups (*n *= 3). (I) Immunofluorescence staining of M1 (CD86, red) and M2 (CD206, green) macrophage markers in spleen sections from the indicated groups, with nuclei counterstained with DAPI (blue). Scale bar: 1000 µm. (J) Quantification of CD86 and CD206 fluorescence intensity from panel I. (K) Relative mRNA expression levels of inflammasome‐priming‐related genes (*Asc*, *Caspase‐1*, *Nlrp3*, and *Iκbα*) in spleen tissues (*n* = 3). (L) Immunoblots analysis of NF‐*κ*B pathway‐related‐proteins (p65, p‐p65, I*κ*B*α*, and p‐I*κ*B*α*) in brain (GAPDH as loading control) and spleen (*β*‐actin as loading control) tissues from the indicated groups. Data are presented as the mean ± SD. Survival curves were compared using the log‐rank (Mantel‐Cox) test. An unpaired 2‐tailed *t*‐test was used in panels (H, K). Statistical significance in panels (E, G, and J) was analyzed using one‐way ANOVA followed by Tukey's multiple comparisons test. ****p < 0.0001, ***p < 0.001, **p < 0.01, *p < 0.05, ^ns^ *p* > 0.05.

Building on the antiparasitic and immunomodulatory effects observed in the zebrafish model, the therapeutic activity of UA‐18*β*GA NPs was further evaluated in a murine model of experimental cerebral malaria induced by *Plasmodium berghei* ANKA (PbA) infection. As shown in Figure 9A, mice were treated intraperitoneally with UA, 18*β*GA, or UA‐18*β*GA NPs (50 mg/kg) for five consecutive days starting at 3 days post‐infection. Compared with the PbA‐infected control group, all treatment groups showed reductions in parasitemia, whereas UA‐18*β*GA NPs produced the strongest effect (Figure 9B). At 8 dpi, the parasitemia in the UA‐18*β*GA NP‐treated group (7.83%) was markedly lower than that in the UA‐ (25.60%) and 18*β*GA‐treated groups (20.14%). Consistently, microscopic examination of blood smears showed fewer infected red blood cells in the UA‐18*β*GA NP‐treated group than in either monomer‐treated group (Figure 9B). This improved parasite control was accompanied by better clinical outcomes. Compared with the infected control group, treatment with UA, 18*β*GA, or UA‐18*β*GA NPs improved survival and attenuated body weight loss, with the nanoparticle‐treated group showing the most favorable overall outcome (Figure 9C,D). In particular, UA‐18*β*GA NPs produced higher survival (∼60%) than either UA (∼50%) or 18*β*GA (∼40%) alone, indicating improved therapeutic efficacy after co‐assembly. The effect of treatment on blood‐brain barrier integrity was then assessed by Evans blue extravasation. Brains from PbA‐infected mice showed marked dye accumulation, whereas reduced Evans blue staining was observed in the UA‐, 18*β*GA‐, and UA‐18*β*GA‐treated groups, with the weakest staining detected in the UA‐18*β*GA NP group. Quantification of Evans blue leakage further supported this observation, showing that UA‐18*β*GA NP treatment reduced vascular leakage more effectively than either monomer treatment (Figure 9E; Figure S12). Histopathological analysis of brain and spleen tissues showed that PbA infection caused marked pathological injury, whereas treatment alleviated these tissue changes to varying extents, with the most pronounced improvement observed in the UA‐18*β*GA NP‐treated group (Figure 9F). In parallel, the expression of the endothelial adhesion molecules *Vcam‐1* and *Icam‐1* was reduced by treatment in both brain and spleen tissues, and the reduction was greater in the UA‐18*β*GA NP group than in the UA or 18*β*GA groups (Figure 9G).

To further evaluate host inflammatory responses, inflammation‐related gene expression in spleen tissue was analyzed. Compared with the PbA‐infected group, UA‐18*β*GA NP treatment more effectively reduced the expression of pro‐inflammatory genes, including *il1β*, *tnfα*, *cd86*, and *inos*, while increasing the expression of anti‐inflammatory or M2‐associated markers, including *il10*, *cd206*, *arg1*, and *tgfβ* (Figure 9H). Immunofluorescence staining of spleen sections further showed lower CD86‐associated signals and higher CD206‐associated signals in the treatment groups relative to the infected control, with the most evident shift observed in the UA‐18*β*GA NP‐treated group (Figure 9I). Quantitative analysis of fluorescence intensity supported these changes in macrophage marker expression (Figure 9J). The expression of genes associated with inflammasome priming was also examined. UA‐18*β*GA NPs reduced the mRNA levels of *Asc*, *Caspase‐1*, and *Nlrp3* while increasing *Iκbα* expression, and these effects were stronger than those observed with UA or 18*β*GA alone (Figure 9K). Western blot analysis further showed that PbA infection increased the phosphorylation of p65 and IκBα in both brain and spleen tissues, whereas treatment reduced pathway activation to different degrees. Among the tested formulations, UA‐18*β*GA NPs showed the strongest reduction in p‐p65 and p‐I*κ*Bα and a corresponding recovery of total I*κ*Bα levels, consistent with a greater inhibitory effect on NF‐*κ*B‐related inflammatory signaling (Figure 9L). Taken together, these results show that UA‐18*β*GA NPs produced stronger therapeutic and immunomodulatory effects than either UA or 18*β*GA alone in experimental cerebral malaria, including better control of parasitemia, improved survival, reduced blood‐brain barrier leakage, alleviated tissue injury, and broader attenuation of inflammatory responses.

## 3 Discussion

Carrier‐free nanodrugs derived from natural products offer an attractive route for improving drug loading and avoiding additional excipients, but their development is still limited by two major issues: the inefficient identification of suitable self‐assembling pairs and the incomplete understanding of the intermolecular interactions associated with assembly and bioactivity [7, 23]. In this study, we combined computational screening with experimental validation to addresses both challenges. Using this workflow, we identified UA and 18*β*GA as a food‐derived molecular pair that co‐assembles into stable nanoparticles with quantitatively supported synergistic antiparasitic activity and host‐protective effects. Importantly, the conclusion of synergy is supported not only by improved efficacy relative to the monomer treatments, but also by accepted reference models, including Bliss and HSA analyses. The positive HSA score (13.076) and the observation that the measured combination activity exceeded the Bliss expected activity across the tested concentrations indicate that the benefit of co‐assembly extends beyond simple additivity.

Natural products are an important source of pharmacologically active compounds, yet their application is often limited by poor aqueous solubility and low bioavailability [33, 34, 35]. Molecular self‐assembly provides one strategy to address these limitations, but the discovery of suitable molecular pairs still relies largely on empirical trial‐and‐error screening [7, 15, 16, 17]. Recent studies have begun to introduce computational tools for this problem, including similarity‐based screening and machine‐learning‐assisted prediction of self‐assembly‐related interactions [8, 9]. Building on these advances, our study integrates 2D structural similarity, 3D pharmacophore matching, docking‐based energetic prioritization, and exploratory RF/SHAP analysis within a single workflow. Rather than claiming a universally generalizable platform, we view this workflow as a practical strategy for prioritizing candidate co‐assembling pairs from chemically complex libraries. In the present study, this approach enabled the prioritization of UA and 18*β*GA, which were subsequently validated experimentally and produced nanoparticles with favorable size distribution, colloidal stability, and sustained release behavior.

A second challenge in carrier‐free nanomedicine is that the intermolecular basis of self‐assembly often remains insufficiently resolved. Spectroscopic approaches such as UV–vis, FTIR, fluorescence, NMR, CD, and XRD can provide important information on the assembled state [24, 25, 26], whereas DFT calculations and MD simulations can further probe interaction patterns and structural evolution [36, 37, 38, 39, 40, 41, 42]. In the present study, these complementary approaches consistently supported the involvement of multiple intermolecular forces in UA‐18*β*GA co‐assembly, including electrostatic complementarity, van der Waals contacts, hydrogen bonding, and hydrophobic association. The additional use of exploratory RF/SHAP analysis provided an interpretable layer linking docking‐related interaction strength with specific molecular descriptors, suggesting that shape complementarity, molecular size matching, polarity/amphiphilicity balance, and hydrogen‐bond‐related features are associated with favorable assembly behavior. Because the RF/SHAP analysis was performed on a limited dataset, it should be regarded as explanatory and hypothesis‐generating rather than broadly predictive; however, it provides a useful bridge between molecular descriptors and experimentally validated assembly outcomes.

A major finding of this work is that UA‐18*β*GA co‐assembly produced advantages beyond formulation improvement alone. In zebrafish parasite assays, the nanoparticles showed synergistic antiparasitic activity, as supported by both Bliss and HSA analyses, together with reduced cytotoxicity relative to the monomers. A recent study defined “meta‐synergistic” drug pairs as combinations that are synergistic both chemically and biologically. By this definition, the UA‐18*β*GA system shows features consistent with meta‐synergy, because co‐assembly provides cooperation at the chemical self‐assembly level, whereas Bliss and HSA analyses support biological synergy in antiparasitic activity. To avoid overstatement, however, we conservatively describe the present findings as synergy rather than formally classifying the system as meta‐synergistic [43]. In mechanistic studies, the antiparasitic effect of UA‐18*β*GA NPs was associated with parasite apoptosis, including mitochondrial membrane potential loss, apoptosis‐related transcriptional changes, and modulation of Erk1/Akt‐associated signaling. At the host level, the nanoparticles also reduced oxidative stress and inflammatory responses, including lower ROS and nitric oxide levels, reduced expression of inflammasome‐priming‐related genes, and changes in macrophage marker expression consistent with an M2‐like shift. In the murine experimental cerebral malaria model, UA‐18*β*GA NPs showed stronger therapeutic and immunomodulatory effects than either monomer treatment alone, including lower parasitemia, improved survival, reduced blood‐brain barrier leakage, alleviated tissue injury, and greater attenuation of NF‐*κ*B‐related inflammatory signaling. These results indicate that the therapeutic advantage of UA‐18*β*GA NPs arises from both direct antiparasitic activity and concurrent modulation of host pathological responses.

Overall, the present findings demonstrate that integrated computational screening can facilitate the identification of self‐assembling natural product combinations and that UA‐18*β*GA NPs represent a promising antiparasitic nanoformulation with synergistic activity and host‐protective effects. Future studies expanding the chemical space and evaluating related systems may further support the broader application of this strategy.

## 4 Conclusion

This work presents an integrated strategy combining computational screening with experimental validation to identify self‐assembling food‐derived molecular combinations with antiparasitic potential. Using this approach, UA and 18*β*GA were identified and shown to self‐assemble into stable nanoparticles. Combined spectroscopic characterization and computational analyses supported the intermolecular interactions associated with nanoparticle formation, while biological evaluation demonstrated synergistic antiparasitic activity together with antioxidant, anti‐inflammatory, and host‐protective effects. These findings highlight UA‐18*β*GA NPs as a promising natural product‐based antiparasitic nanoformulation and support the use of integrated screening approaches for the discovery of self‐assembling bioactive molecular combinations.

## 5 Experimental Section

### 5.1 Parasites, Cells, and Chemical Reagents

The *I. multifiliis* isolate used in this study was the G5 strain. The parasite was maintained by serial passage in specific pathogen‐free (SPF) fish at 22 ± 1 °C. The Epithelioma papulosum cyprini (EPC) cell line was provided by the National Experimental Cell Resource Sharing Platform of China and maintained in accordance with the Chinese agricultural industry standard SC/T 7016.5‐2012. EPC cells were cultured in minimum essential medium (MEM) supplemented with 10% fetal bovine serum (FBS) and 1% penicillin‐streptomycin solution at 25 °C. The *Plasmodium berghei* ANKA (PbA) strain was used for the experimental cerebral malaria (ECM) model and maintained by serial passage in C57BL/6 mice.

Ursolic acid (UA; Aladdin, Cat. No. U118635, purity ≥ 98%) was purchased from Aladdin, whereas 18*β*‐glycyrrhetinic acid (18*β*GA; Sigma‐Aldrich, Cat. No. G10105, purity ≥ 97%), albendazole (ABZ; Sigma‐Aldrich, Cat. No. A4673, purity ≥ 98%), methanol (Sigma‐Aldrich, Cat. No. 34860, purity ≥ 99.9%), deionized formamide (Sigma‐Aldrich, Cat. No. S4117), phosphate‐buffered saline (PBS; Sigma‐Aldrich, Cat. No. P4474), and Evans blue (Sigma‐Aldrich, Cat. No. E2129) were purchased from Sigma‐Aldrich.

### 5.2 Preparation of UA‐18*β*GA Self‐Assembled Nanoparticles

UA‐18*β*GA NPs were prepared by an antisolvent precipitation method. Briefly, UA and 18*β*GA were dissolved in methanol at an equimolar ratio (1:1; 1 mM each) to obtain a homogeneous stock solution. Then, 1 mL of the methanolic solution was added dropwise into 5 mL of deionized water at a rate of approximately 1 drop every 2 s under continuous magnetic stirring at 300 rpm and 60 °C. The resulting dispersion was stirred for 60 min to promote molecular self‐assembly and nanoparticle formation. After cooling to room temperature, the suspension was transferred into a dialysis bag (MWCO 3.5 kDa) and dialyzed against deionized water for 24 h to remove residual methanol and non‐assembled molecules. The dialysis medium was replaced six times, with more frequent water changes during the initial stage (at 0.5, 1, 2, and 4 h), followed by additional changes at 8 and 16 h. After dialysis, the resulting suspension was filtered through filter paper to remove precipitated aggregates, and the filtrate was then lyophilized to obtain UA‐18*β*GA NPs as a dry powder. Because UA‐18*β*GA NPs are carrier‐free and consist only of UA and 18*β*GA without any inert excipient, the concentrations reported for the nanoparticle formulation in the following biological experiments refer to the total concentration of active components in the co‐assembled system.

### 5.3 Optimization of the Preparation Conditions for UA‐18*β*GA NPs

To optimize the preparation conditions for UA‐18*β*GA NPs, a stepwise screening procedure was performed. First, the initial drug concentration was preliminarily evaluated at 0.1, 1, and 10 mM. After self‐assembly, the dispersion prepared at 1 mM appeared clearer, exhibited a more obvious Tyndall effect, and showed the highest drug loading; therefore, 1 mM was selected as the starting concentration for subsequent optimization. Next, the molar ratio of UA to 18*β*GA was optimized under fixed preparation conditions, including an assembly temperature of 60 °C, a stirring duration of 60 min, and a stirring speed of 300 rpm. Nanoparticles were prepared according to the antisolvent precipitation procedure described above, and the resulting formulations were evaluated based on particle morphology, hydrodynamic diameter, zeta potential, and drug loading. Using these criteria, the optimal UA/18*β*GA molar ratio was determined to be 1:1. After the molar ratio was fixed at 1:1, the preparation temperature was further optimized to determine whether 60 °C was the most suitable condition. Subsequently, under the optimized molar ratio (1:1) and temperature (60 °C), the assembly time was further screened. Among the tested conditions, a preparation time of 60 min produced nanoparticles with the most favorable morphology and particle size characteristics. Based on the combined results, the optimal preparation conditions for UA‐18*β*GA NPs were determined to be an initial concentration of 1 mM, a UA/18*β*GA molar ratio of 1:1, an assembly temperature of 60 °C, and a stirring duration of 60 min.

### 5.4 Characterization of UA‐18*β*GA NPs

The average particle size, polydispersity index (PDI), and zeta potential of UA‐18*β*GA NPs in aqueous suspension were determined by dynamic light scattering (DLS), with all measurements performed in triplicate. UV–vis absorption spectra of aqueous dispersions of UA‐18*β*GA NPs, UA, and 18*β*GA were recorded using a NanoDrop One spectrophotometer (Thermo Fisher Scientific, USA) with deionized water as the reference. Measurements were conducted in a 10 mm quartz cuvette at 25 °C with a spectral resolution of 1 nm over the range of 190–500 nm. Fluorescence emission spectra of UA‐18*β*GA NPs, UA, and 18*β*GA in methanol were measured at 25 °C using a fluorescence spectrophotometer (RF‐6000, Shimadzu, Japan). Samples were excited at 260 nm, and emission spectra were collected over the range of 300–500 nm with a spectral resolution of 2 nm. All fluorescence measurements were performed in triplicate. Fourier‐transform infrared (FTIR) spectra were acquired using a Bruker VERTEX 70v spectrometer (Germany). Samples were mixed with KBr at a mass ratio of 1:100 and compressed into pellets. Spectra were recorded in transmission mode over the range of 4000–400 cm^−^^1^ at a resolution of 4 cm^−1^, using a pure KBr pellet as the background. X‐ray powder diffraction (XRD) patterns were collected using a Bruker D8 ADVANCE A25 diffractometer (Germany) equipped with a graphite monochromator and Cu K*α* radiation (*λ* = 1.5406 Å), operated at 40 kV and 40 mA. Data were acquired over a 2θ range of 5°–50° with a step size of 0.01°. ^1^H NMR spectra were recorded on a Bruker Avance 400 MHz spectrometer (Germany). Samples were dissolved in CDCl_3_ (1.0 mL) and analyzed at 298 K. Circular dichroism (CD) spectra were obtained using an Applied Photophysics Chirascan V100 spectropolarimeter (UK). Measurements were performed at room temperature in a quartz cuvette with a path length of 1 mm over the wavelength range of 190–350 nm. The CD Data were processed and analyzed using CDNN Deconvolution Software (version 2.0).

### 5.5 Similarity‐Based Screening With RDKit

Structural similarity between compounds in the food‐derived compound database and the reference molecules (ABA and OA) was calculated using RDKit. Morgan fingerprints (radius = 2, nBits = 2048) were generated for all compounds, and pairwise Tanimoto coefficients were computed to quantify molecular similarity. Candidate compounds were ranked according to their similarity scores relative to the reference molecules, and those with higher similarity were considered to have greater potential for self‐assembly.

### 5.6 Pharmacophore Matching and Screening

To identify molecules with high self‐assembly potential, ligand‐based pharmacophore models of OA and ABA were generated using the ligand‐based pharmacophore modeling module in LigandScout 4.5 (Ligand GmbH, Austria). A screening database containing food‐derived compounds was established using the built‐in database construction module. Pharmacophore matching was then carried out using the pharmacophore‐fit scoring function. The screening mode was set to match all query features, with stop after first matching conformation enabled, the maximum number of omitted features set to 0, the minimum number of required features set to 3, and exclusion volumes included in the screening process. Potential self‐assembling molecules were screened and ranked according to their pharmacophore fit scores.

### 5.7 Random Forest (RF) and SHapley Additive exPlanations (SHAP) Analysis

An exploratory machine learning analysis was performed to identify molecular features associated with strong intermolecular binding and potential self‐assembly‐driving interactions. Six predefined molecular features were extracted, and docking‐derived binding affinity values between six reference molecules and one candidate molecule were used as the response variable. A RF regression model was implemented in Python using scikit‐learn, with 200 trees, a maximum tree depth of 5, a minimum of 3 samples required for node splitting, a minimum of 2 samples required at each leaf node, and a fixed random seed of 42; all other parameters were kept at their default settings. Because of the limited sample size, the model was used primarily for exploratory interpretation rather than predictive modeling. Feature importance analysis was performed to rank the relative contributions of individual molecular features to binding affinity. SHAP analysis was further conducted to evaluate the magnitude and direction of each feature's contribution to the predicted binding affinity. Features that consistently reduced the predicted binding energy were considered key structural determinants favoring molecular self‐assembly.

### 5.8 Molecular Dynamics (MD) Simulation

For the self‐assembly MD simulations, the molecular structures were first optimized using DFT‐D3/DMol^3^ (Biovia, USA). The CHARMM36 force field was used to generate molecular topology files, and the TIP3P water model was employed to construct a solvated system in a cubic box of 6 × 6 × 6 nm^3^. The system contained 20 UA molecules, 20 18*β*GA molecules, and 6000 TIP3P water molecules. The protonation states of all molecules were assigned to represent neutral pH conditions, and the system was set with zero net charge and without the addition of counterions. Energy minimization was performed using GROMACS 2020.6 (University of Groningen, the Netherlands) with the steepest descent algorithm. The minimization parameters were set as follows: integrator = steep, convergence criterion emtol = 1000 kJ/mol/nm, initial step size emstep = 0.01 nm, maximum steps nsteps = 50000; the conjugate gradient method was applied if convergence was not achieved. This was followed by equilibration under periodic boundary conditions, including 500 ps in the NVT ensemble and 500 ps in the NPT ensemble, and then by a 500 ns production run at 333 K with a time step of 2 fs. Bonds involving hydrogen atoms were constrained using the LINCS algorithm. Trajectory visualization and analysis were performed using VMD 1.9.3 (University of Illinois Urbana‐Champaign, USA).

### 5.9 Molecular Docking

Molecular structures were obtained from ChemSpider and preprocessed by hydrogen addition and conformational refinement in Avogadro 1.95. Subsequent geometry optimization was performed using the DMol^3^ module in Materials Studio 2019 (Biovia, USA). The optimization task was set to geometry optimization with the calculation quality defined as medium. The generalized gradient approximation (GGA) with the Perdew–Burke–Ernzerhof (PBE) functional was used, and all calculations were carried out using the DNP basis set. The convergence criteria were set according to the medium‐quality requirements, with a maximum energy change of ≤2.0 × 10^−5^ Ha, a maximum force of ≤0.004 Ha/Å, and a maximum displacement of ≤0.005 Å. Docking simulations and binding affinity predictions were performed using AutoDock Vina 1.2.0 (Scripps Research, USA). The five top‐scoring conformations with the lowest predicted binding energies were retained for intermolecular interaction analysis, and all conformations were visualized using PyMOL 2.4 (DeLano Scientific LLC, USA).

### 5.10 Density Functional Theory (DFT) Calculations

The geometric and electronic properties of UA and 18*β*GA were investigated by quantum chemical calculations using Gaussian 16 Rev. C.01. The molecular structures of UA and 18*β*GA were fully optimized at the B3LYP/6‐311G(d,p) level of theory, with the SMD implicit solvation model used to simulate an aqueous environment. Electrostatic potential (ESP) surfaces were generated in GaussView 6.0 based on optimized wavefunctions and mapped in the range of −0.02 to 0.02 a.u. (red, electron‐rich; blue, electron‐deficient). Frontier molecular orbital (FMO) energy levels, including the highest occupied molecular orbital (HOMO) and lowest unoccupied molecular orbital (LUMO), were calculated at the same B3LYP/6‐311G(d,p) level, and the HOMO‐LUMO energy gap (Δ*E*) was calculated as Δ*E* = *E*_LUMO_ − *E*_HOMO_ to evaluate chemical reactivity. Weak interactions including hydrogen bonds, van der Waals forces, and other weak dispersive interactions, were analyzed using Multiwfn 3.8 through the non‐covalent interaction (NCI) method based on the reduced density gradient (RDG). The electron density threshold (*ρ*) was set to 0.05 a.u., and the gradient norm threshold (*g*) was set to 0.02 a.u. NCI isosurfaces were used to distinguish different interaction types: blue indicated attractive interactions, green represented van der Waals interactions, and red denoted repulsive interactions. Weak interaction diagrams were visualized using VMD 1.9.3.

### 5.11 in vitro Release of UA‐18*β*GA NPs

The in vitro release behavior of free UA and UA‐18*β*GA NPs was investigated using a dialysis method. Briefly, 2 mL of each sample (1000 mg/L) was placed in a dialysis bag (molecular weight cutoff: 3 kDa), with the internal phase containing of the same corresponding buffer system as the external release medium and supplemented with 10% DMSO as a cosolvent. The dialysis bags were immersed in 20 mL of release medium, consisting of either acetate buffer (pH 5.4) or PBS (pH 7.4), and maintained at 25°C under continuous stirring at 200 rpm. At predetermined time points (0.5, 1, 2, 4, 6, 12, 24, 48, 72, 96, and 120 h), 2 mL aliquots were withdrawn from the external medium and immediately replaced with an equal volume of fresh corresponding medium. The amount of released compound was quantified by HPLC, and the release rate was expressed as the cumulative release percentage.

### 5.12 In Vivo Distribution

The in vivo biodistribution of UA‐18*β*GA NPs in zebrafish was evaluated using a noninvasive live imaging system. Zebrafish were orally administered DiR‐labeled UA‐18*β*GA NPs at a dose of 20 mg/kg, with free DiR dye used as the control. At predetermined time points (6, 12, 24, 36, and 48 h) after administration, the zebrafish were anesthetized and imaged using an IVIS Spectrum imaging system under identical acquisition setting. Fluorescence signals were collected and quantified within defined regions of interest (ROIs) to compare the tissue distribution and accumulation kinetics of the nanoparticles with those of the free dye control. Background fluorescence was subtracted prior to quantitative analysis.

### 5.13 Safety Assessments of UA‐18*β*GA NPs

**Embryotoxicity**: Zebrafish embryo toxicity assays were performed in accordance with OECD guideline 236. Prior to exposure, viable and healthy embryos with normal morphology were selected for the experiment. Thirty fertilized embryos were added to each well of a 12‐well plate and exposed to UA, 18*β*GA, or UA‐18*β*GA NPs solutions in oxygenated distilled water (final volume: 5 mL; concentrations: 1, 2, and 3 mg/L). Embryonic development was monitored at 0, 24, 48, 72 and 96 h using a microscope (Olympus BX41, Japan). All embryos in the control group hatched successfully by the end of the exposure period.

**Acute toxicity**: Groups of 20 zebrafish (3.0–3.5 cm) were exposed to UA, 18*β*GA, and UA‐18*β*GA at concentrations of 2, 4, 6, and 8 mg/L in static renewal systems, with untreated fish used as controls. Mortality was recorded at 6 h intervals from the start of exposure. Survival rates were determined after 96 h, and survival curves were plotted using GraphPad Prism (GraphPad software, USA).

### 5.14 Hemolysis Assay

Hemocompatibility was evaluated using erythrocytes isolated from largemouth bass (*Micropterus salmoides*). Red blood cells (RBCs) were washed three times with normal saline, and 300 µL of the washed RBC suspension was incubated with serially diluted compounds (UA, 18*β*GA, and UA‐18*β*GA; 2, 4, 6, 8, 10, 12 mg/L) at 25 °C for 4 h. After centrifugation at 3000 rpm for 5 min, 200 µL of the supernatant was collected, and hemoglobin release was measured spectrophotometrically at 545 nm using a multifunctional microplate reader (BioTek Synergy H1, USA). Two control groups were included: a negative control (normal saline, 0% hemolysis) and a positive control (distilled water, 100% hemolysis). The hemolysis rate was calculated according to the following equation:

$$\mathrm{Hemolysis}\;\mathrm{rate}\left(\%\right)=\left(OD_{\mathrm{sample}}-OD_{\mathrm{negative}}\right)/\left(OD_{\mathrm{Positive}}-OD_{\mathrm{negative}}\right)\times 100\%$$

### 5.15 Antiparasitic Efficacy Evaluation in Zebrafish

The antiparasitic efficacy of the UA‐18*β*GA NPs against *I. multifiliis* was systematically evaluated at three distinct developmental stages. For the theront stage, approximately 300 freshly released theronts were exposed to different concentrations of the test compounds in 96‐well plates, with albendazole used as the positive control and free UA and 18*β*GA used as reference controls. Viability was assessed after 4 h of incubation at 22 ± 0.5 °C. For the tomont stage, 30 protomonts per well were allowed to encyst in 24‐well plates for 6 h before treatment with the compounds, with albendazole (ABZ) included as the positive control, and theront release and tomont mortality were recorded after 18 h. For the trophont stage, infected zebrafish (≥20 trophonts per fish) were exposed to compounds (0.5–1.0 mg/L) in beaker systems, and detached parasites were collected and monitored for theront release over 22 h. All experiments were performed in triplicate under controlled temperature conditions (22 ± 0.5 °C), and parasite viability and development were evaluated microscopically using an Olympus BX41 microscopy (Japan).

### 5.16 Synergy Analysis of Cytotoxicity and Antiparasitic Activity

To evaluate the interaction between UA and 18*β*GA, matrix‐based combination assays were performed for both EPC cell viability and antiparasitic activity. For cytotoxicity analysis, EPC cells were treated with UA at 4, 8, 16, 32, and 64 mg/L in combination with 18*β*GA at 2, 4, 8, 16, and 32 mg/L. Cell viability was measured using a CCK‐8 assay at 450 nm (Beyotime, Cat. Nos. C0037). For antiparasitic synergy analysis, *I. multifiliis* was treated with matrix combinations of UA and 18*β*GA at 0.1, 0.2, 0.3, 0.4, and 0.5 mg/L, and antiparasitic activity was assessed using the in vitro assay described above. The resulting dose‐response matrices were analyzed using SynergyFinder+ [44]. Bliss independence and HSA models were applied to evaluate antiparasitic synergy, whereas the ZIP model was used to analyze the combined cytotoxic effects on EPC cells.

### 5.17 Evaluation of Therapeutic Efficacy in ECM Mice

All animal procedures were performed in accordance with protocols approved by the Institutional Animal Care and Use Committee (IACUC) of Northwest A&F University, China (Authorization No. NWAFU‐314020038). Male C57BL/6 mice (6 weeks old, 17.9–19.3 g) were intraperitoneally inoculated with 4.2 × 10^6^ *Plasmodium berghei* ANKA (PbA)‐infected red blood cells collected from a donor mouse with approximately 37% parasitemia. Starting on day 3 post‐infection, mice in the treatment groups (*n* = 5 per group) received daily intraperitoneal injections of either UA or UA‐18*β*GA NPs (50 mg/kg) for five consecutive days. Mice in the vehicle control group received an equivalent volume of PBS. Parasitemia was monitored daily throughout the study by microscopic examination of Giemsa‐stained thin blood smears. On day 8 post‐infection, each mouse was intravenously injected with 100 µL of a 2% (w/v) Evans blue in PBS (pH 7.4). After circulation for 1 h, the mice were euthanized and transcardially perfused with 15 mL PBS. The brains were then carefully excised and imaged. Subsequently, each brain was incubated in 2 mL deionized formamide at 37 °C for 48 h to extract the dye. Evans blue concentration in the formamide extract was determined by measuring the absorbance of 100 µL of the supernatant at 630 nm using a microplate reader (BioTek Synergy H1, USA).

### 5.18 Histopathological Analysis

Freshly isolated tissue samples from zebrafish (muscle, gill, and liver) or mice (brain) were immediately fixed in ice‐cold 4% paraformaldehyde (PFA), embedded, sectioned into 4 µm slices, and stained with hematoxylin and eosin (H&E) according to standard protocols. Histopathological images were obtained under bright‐field illumination using a light microscope (Olympus BX53, Japan).

### 5.19 Immunofluorescence Staining

Tissue samples from mice (spleen) and zebrafish (gill) were fixed, paraffin‐embedded, and sectioned into 4 µm slices. After deparaffinization and antigen retrieval, the sections were incubated with primary antibodies against CD86 (1:100, Novus Biologicals, Littleton, CO, USA) and CD206 (1:500, Abcam, Cambridge, UK), followed by corresponding fluorophore‐conjugated secondary antibodies. Cell nuclei were counterstained with DAPI. Whole‐slide fluorescence images was acquired using a high‐resolution slide scanner under identical imaging settings for all samples.

### 5.20 Quantitative Real‐Time PCR (qPCR)

Following treatment with UA, 18*β*GA, and UA‐18*β*GA NPs, total RNA was extracted from parasite and zebrafish samples using the TRIzol method. First‐strand cDNA was synthesized using the Hiscript III Reverse Transcriptase Kit (Vazyme Biotech, China). Quantitative real‐time PCR was performed on a Gene‐9660 system (Genestar Biosolutions) using SYBR Green Master Mix. The amplification program consisted of an initial denaturation at 95 °C for 30 s, followed by 40 cycles of 95 °C for 10 s and 60 °C for 30 s. Melting curve analysis was performed at 95 °C for 15 s, 60 °C for 60 s, and 95 °C for 15 s. All reactions were performed in triplicate, and the primer sequences are listed in Tables S1–S6. Gene expression levels were normalized to *β*‐actin and calculated using the 2^−ΔΔCt^ method.

### 5.21 RNA Sequencing Analysis

Total RNA was extracted from parasite samples using Trizol reagent (Thermo Fisher Scientific, Cat. No. 15596018). Samples included the untreated group (ddH_2_O; C1, C2, and C3) and the UA‐18*β*GA NP‐treated group (0.5 mg/L for 6 h; T1, T2, and T3). RNA integrity was assessed using an Agilent 5300 Fragment Analyzer, and samples with an RNA integrity number (RIN) >7.0 were used for library construction. Strand‐specific cDNA libraries were prepared using the mRNA Capture Beads 2.0 system (Yeasen) and sequenced on an Illumina Novaseq X Plus platform with a 2 × 150 bp paired‐end configuration by LC‐Bio (Hangzhou, China). Raw sequencing data were processed by FastQC (v0.10.1) for quality assessment, HISAT2 (v2.2.1) for alignment to the reference genome (GCF_000220395.1), and StringTie (v2.1.6) for transcript assembly and expression quantification. Differential expression analysis was performed using DESeq2 (v1.22.2), and genes with |log_2_FC| ≥ 1 and *p* < 0.05 were considered differentially expressed. Alternative splicing events were identified using rMATS (v4.1.1). Functional enrichment analyses were performed based on the GO (2021‐05) and KEGG (2021‐05) databases. All bioinformatics analyses were conducted in R (v3.6).

### 5.22 Western Blot Analysis

Mouse brain and spleen tissues were rapidly collected, snap‐frozen in liquid nitrogen, and stored until further use. Equal amounts of tissue were lysed in Western and IP lysis buffer (Beyotime, China) supplemented with phosphatase inhibitor, protease inhibitor, and PMSF. Protein concentrations were quantified using the BCA protein assay. Equal amounts of protein were separated by SDS‐PAGE and transferred onto polyvinylidene difluoride (PVDF) membranes. After blocking, the membranes were incubated with primary antibodies against p‐p65 (Bioss, Cat. No. bs‐5661R, 1:1000), p65 (Bioss, Cat. No. bs‐20160R, 1:2000), I*κ*B*α* (Proteintech, Cat. No. 66418‐1‐Ig, 1:10000), and p‐I*κ*B*α* (Proteintech, Cat. No. 68999‐1‐Ig, 1:5000). GAPDH (Proteintech, Cat. No. 60004‐1‐Ig, 1:100000) was used as the loading control for brain tissue, whereas *β*‐actin (Proteintech, Cat. No. 66009‐1‐Ig, 1:50000) was used as the loading control for spleen tissue. The membranes were then incubated with horseradish peroxidase (HRP)‐conjugated secondary antibodies (Beyotime, Cat. Nos. A0208 and A0216; 1:500) for 1 h, followed by three washes with TBST for 10 min each. Protein bands were visualized using an enhanced chemiluminescence (ECL) detection system and quantified using ImageJ software.

### 5.23 Statistical Analyses

Normality was assessed using the Shapiro–Wilk test. Continuous variables are presented as mean ± standard deviation (SD). Comparisons among three or more groups were performed using one‐way analysis of variance (ANOVA), followed by Tukey's multiple comparisons test. Comparisons between two groups were conducted using an unpaired two‐tailed Student's *t*‐test. Survival data were analyzed using the Kaplan–Meier method, and differences among groups were assessed using the log‐rank (Mantel–Cox) test. All statistical analyses were performed using SPSS version 20.0 (IBM Corp., Armonk, NY, USA) and GraphPad Prism (GraphPad Software, USA).

## Author Contributions

S.Y.Q., T.W., G.X.W., and F.L. conceived and designed the study. S.Y.Q., T.W., J.T.L., J.C.Q., B.Y., Y.H.L., and P.F.L. performed the experiments and collected the data. S.Y.Q., T.W., J.T.L., and J.C.Q. analyzed the data. S.Y.Q. and T.W. drafted the manuscript. G.X.W. and F.L. supervised the project, revised the manuscript, and secured funding. All authors discussed the results and approved the final manuscript.

## Conflicts of Interest

The authors declare no conflicts of interest.

## Supporting information




**Supporting File 1**: advs75363‐sup‐0001‐SuppMat.docx.

**Supporting File 2**: advs75363‐sup‐0002‐DataFile.zip.

## Data Availability

The data that support the findings of this study are available in the Supporting Information of this article.

## References

[1] L. H. Liu and X. Z. Zhang , “Carrier‐Free Nanomedicines for Cancer Treatment,” Progress in Materials Science 125 (2022): 100919, 10.1016/j.pmatsci.2021.100919.

[2] H. Mei , S. S. Cai , D. Huang , H. L. Gao , J. Cao , and B. He , “Carrier‐Free Nanodrugs with Efficient Drug Delivery and Release for Cancer Therapy: from Intrinsic Physicochemical Properties to External Modification,” Bioactive Materials 8 (2022): 220. 34541398 10.1016/j.bioactmat.2021.06.035 PMC8424425

[3] Y. X. Liu , L. L. Wang , L. Zhao , Y. G. Zhang , Z. T. Li , and F. H. Huang , “Multiple Hydrogen Bonding Driven Supramolecular Architectures and Their Biomedical Applications,” Chemical Society Reviews 53 (2024): 1592–1623, 10.1039/D3CS00705G. 38167687

[4] J. Y. Liu , Y. N. Bai , Y. G. Li , X. L. Li , and K. Luo , “Reprogramming the Immunosuppressive Tumor Microenvironment through Nanomedicine: An Immunometabolism Perspective,” EBioMedicine 107 (2024): 105301, 10.1016/j.ebiom.2024.105301. 39178747 PMC11388279

[5] X. Li , Z. Duan , X. Chen , et al., “Impairing Tumor Metabolic Plasticity via a Stable Metal‐Phenolic‐Based Polymeric Nanomedicine to Suppress Colorectal Cancer,” Advanced Materials 35 (2023): 2300548, 10.1002/adma.202300548. 36917817

[6] H. J. Jin , L. Q. Wang , and R. Bernards , “Rational Combinations of Targeted Cancer Therapies: Background, Advances and Challenges,” Nature Reviews Drug Discovery 22 (2023): 213–234, 10.1038/s41573-022-00615-z. 36509911

[7] X. Guo , W. Luo , L. Wu , et al., “Natural Products from Herbal Medicine Self‐Assemble into Advanced Bioactive Materials,” Advanced Science 11 (2024): 2403388, 10.1002/advs.202403388. 39033533 PMC11425287

[8] Y. M. Shan , Z. M. Zhang , H. L. Zhou , et al., “Deep Learning‐Driven Co‐Assembly of Naturally Sourced Compound Nanoparticles for Potentiated Cancer Immunotherapy,” Advanced Functional Materials 36 (2025): 19567.

[9] B. Fang , F. Pan , T. Shan , et al., “An Integrated Virtual Screening Platform to Identify Potent Co‐Assembled Nanodrugs for Cancer Treatment,” Advanced Materials 37 (2025): 2414154, 10.1002/adma.202414154. 39988868

[10] Y. Shamay , J. Shah , M. Isik , et al., “Quantitative Self‐Assembly Prediction Yields Targeted Nanomedicines,” Nature Materials 17 (2018): 361–368, 10.1038/s41563-017-0007-z. 29403054 PMC5930166

[11] F. Li , J. Han , T. Cao , and L. X. Li , “Design of Self‐Assembly Dipeptide Hydrogels and Machine Learning via Their Chemical Features,” Proceedings of the National Academy of Sciences USA 116 (2019): 11259–11264, 10.1073/pnas.1903376116. PMC6561259 31110004

[12] D. Reker , Y. Rybakova , A. R. Kirtane , et al., “Computationally Guided High‐Throughput Design of Self‐Assembling Drug Nanoparticles,” Nature Nanotechnology 16 (2021): 725–733, 10.1038/s41565-021-00870-y. PMC8197729 33767382

[13] M. Peng , Q. Peng , W. Li , et al., “Atomic Insights into Self‐Assembly of Zingibroside R1 and Its Therapeutic Action against Fungal Diseases,” Advanced Materials 37 (2025): 2503283, 10.1002/adma.202503283. 40326238

[14] R. Zhang , L. Guo , Q. Li , et al., “Biodegradable Carrier‐Free Nanomedicine via Self‐Assembly of Pure Drug Molecules for Triple Sensitization of Radiotherapy,” ACS Nano 19 (2025): 16355–16371, 10.1021/acsnano.4c15736. 40265972

[15] Q. Zhao , Y. Li , Q. Sun , et al., “Self‐Assembled Genistein Nanoparticles Suppress the Epithelial‐Mesenchymal Transition in Glioblastoma by Targeting Mmp9,” Materials Today Bio 31 (2025): 101606, 10.1016/j.mtbio.2025.101606. PMC11919400 40104644

[16] J. Zeng , Y. Zhang , Y. Gao , et al., “Biomimetic Ginsenoside Rb1 and Probucol Co‐Assembled Nanoparticles for Targeted Atherosclerosis Therapy via Inhibition of Oxidative Stress, Inflammation, and Lipid Deposition,” ACS Nano 19 (2025): 22968–22987, 10.1021/acsnano.5c02492. 40534137

[17] Y. Li , Y. D. Zhou , Y. Q. Tan , and G. Deng , “Anti‐Inflammatory Treatment of Subarachnoid Hemorrhage by Self‐Assembled Silymarin Nanoparticles,” Small Science 5 (2025): 2400322, 10.1002/smsc.202400322. 40657200 PMC12244996

[18] Y. B. Fan , J. Ma , Y. R. Li , X. Y. Huang , S. G. Feng , and D. Y. Chen , “Co‐Assembly of Synthetic Particles with Heterogenous Components,” Chemistry of Materials 36 (2024): 4011–4033, 10.1021/acs.chemmater.4c00134.

[19] J. Zhang , L. Xiang , B. Zhou , et al., “Precision Self‐Assembly of Supramolecules with Heterogeneous Derivatives,” Advanced Functional Materials 34 (2024): 2410997, 10.1002/adfm.202410997.

[20] K. Kim , A. Jang , H. Shin , et al., “Concurrent Optimizations of Efficacy and Blood–Brain Barrier Permeability in New Macrocyclic LRRK2 Inhibitors for Potential Parkinson's Disease Therapeutics,” Journal of Medicinal Chemistry 67 (2024): 7647–7662, 10.1021/acs.jmedchem.4c00520. 38684226

[21] K. Puls , A.‐L. Olivé‐Marti , S. Hongnak , D. Lamp , M. Spetea , and G. Wolber , “Discovery of Novel, Selective, and Nonbasic Agonists for the Kappa‐Opioid Receptor Determined by Salvinorin A‐Based Virtual Screening,” Journal of Medicinal Chemistry 67 (2024): 13788–13801, 10.1021/acs.jmedchem.4c00590. 39088801 PMC11345774

[22] J.‐L. Yu , C. Zhou , X.‐L. Ning , et al., “Knowledge‐Guided Diffusion Model for 3D Ligand‐Pharmacophore Mapping,” Nature Communications 16 (2025): 2269, 10.1038/s41467-025-57485-3. PMC11885826 40050649

[23] H. Liu , X. Jin , S. Liu , et al., “Recent Advances in Self‐Targeting Natural Product‐Based Nanomedicines,” Journal of Nanobiotechnology 23 (2025): 31, 10.1186/s12951-025-03092-9. 39833846 PMC11749302

[24] Y. Han , X. Yang , S. Fu , et al., “Co‐Assembly of Abietic Acid and Oleanolic Acid into Nanoparticles Encapsulating Proanthocyanidin B2 to Improve Pac B2 Bioavailability and Thermal Stability,” Food Chemistry 485 (2025): 144512, 10.1016/j.foodchem.2025.144512. 40315762

[25] Y. Xu , Z. Chen , W. Hao , et al., “Berberine and Magnolol Exert Cooperative Effects on Ulcerative Colitis in Mice by Self‐Assembling into Carrier‐Free Nanostructures,” Journal of Nanobiotechnology 22 (2024): 538, 10.1186/s12951-024-02804-x. 39227962 PMC11373475

[26] S. Gao , H. Zheng , S. Xu , et al., “Novel Natural Carrier‐Free Self‐Assembled Nanoparticles for Treatment of Ulcerative Colitis by Balancing Immune Microenvironment and Intestinal Barrier,” Advanced Healthcare Materials 12 (2023): 2301826, 10.1002/adhm.202301826. 37681364

[27] V. Hassija , V. Chamola , A. Mahapatra , et al., “Interpreting Black‐Box Models: A Review on Explainable Artificial Intelligence,” Cognitive Computation 16 (2024): 45–74, 10.1007/s12559-023-10179-8.

[28] Y. Nohara , K. Matsumoto , H. Soejima , and N. Nakashima , “Explanation of Machine Learning Models Using Shapley Additive Explanation and Application for Real Data in Hospital,” Computer Methods and Programs in Biomedicine 214 (2022): 106584, 10.1016/j.cmpb.2021.106584. 34942412

[29] H. H. Nguyen , J. L. Viviani , and S. B. Jabeur , “Bankruptcy Prediction Using Machine Learning and Shapley Additive Explanations,” Review of Quantitative Finance and Accounting 65 (2025): 107–148, 10.1007/s11156-023-01192-x.

[30] J. D. Okombo and A. Fidock , “Towards Next‐Generation Treatment Options to Combat *Plasmodium Falciparum* Malaria,” Nature Reviews Microbiology 23 (2025): 178–191, 10.1038/s41579-024-01099-x. 39367132 PMC11832322

[31] L. Ochola , J. Gitaka , B. Kanoi , et al., “Malaria Chemotherapeutics What Next for Africa,” Advanced Therapeutics 8 (2025): 2400453, 10.1002/adtp.202400453.

[32] L. Wang , X. C. Zhu , H. L. Liu , and B. G. Sun , “Medicine and Food Homology Substances: A Review of Bioactive Ingredients, Pharmacological Effects and Applications,” Food Chemistry 463 (2025): 141111, 10.1016/j.foodchem.2024.141111. 39260169

[33] A. G. Atanasov , S. B. Zotchev , V. M. Dirsch , and C. T. Supuran , “Natural Products in Drug Discovery: Advances and Opportunities,” Nature Reviews Drug Discovery 20 (2021): 200–216, 10.1038/s41573-020-00114-z. 33510482 PMC7841765

[34] S. B. Bharate and C. W. Lindsley , “Natural Products Driven Medicinal Chemistry,” Journal of Medicinal Chemistry 67 (2024): 20723–20730, 10.1021/acs.jmedchem.4c02736. 39629819

[35] C. Qiu , J. Z. Zhang , B. Wu , et al., “Advanced Application of Nanotechnology in Active Constituents of Traditional Chinese Medicines,” Journal of Nanobiotechnology 21 (2023): 456, 10.1186/s12951-023-02165-x. 38017573 PMC10685519

[36] L. Zong , Y. Dai , J. Xu , et al., “Luteolin and Glycyrrhetinic Exert Cooperative Effect on Liver Cancer by Selfassembling into Carrier‐free Nanostructures,” Chinese Chemical Letters 36 (2025): 111325, 10.1016/j.cclet.2025.111325.

[37] H. Jiang , Q. Lu , X. Huang , et al., “Sinomenine‐Glycyrrhizic Acid Self‐Assembly Enhanced the Anti‐Inflammatory Effect of Sinomenine in the Treatment of Rheumatoid Arthritis,” Journal of Controlled Release 382 (2025): 113718, 10.1016/j.jconrel.2025.113718. 40220871

[38] L. Xia , Q. Yang , K. Fu , et al., “Unveiling the Renoprotective Mechanisms of Self‐Assembled Herbal Nanoparticles from *Scutellaria Barbata* and *Scleromitrion Diffusum* in Acute Kidney Injury: A Nano‐Tcm Approach,” Acta Pharmaceutica Sinica B 15 (2025): 4265–4284, 10.1016/j.apsb.2025.05.024. 40893687 PMC12399204

[39] T. Liang , Y. Wu , Q. Zeng , et al., “Development of a Self‐Assembled Micelles Based on Cryptotanshinone and Glycyrrhizic Acid: An Efficient Strategy for Acne Treatment,” International Journal of Pharmaceutics 674 (2025): 125411, 10.1016/j.ijpharm.2025.125411. 40020947

[40] W. Qian , B. Zhang , M. Gao , et al., “Supramolecular Prodrug Inspired by the Rhizoma Coptidis‐Fructus Mume Herbal Pair Alleviated Inflammatory Diseases by Inhibiting Pyroptosis,” Journal of Pharmaceutical Analysis 15 (2025): 101056, 10.1016/j.jpha.2024.101056. 39974618 PMC11835567

[41] P. Pang , W. Liu , S. Ma , et al., “Self‐Assembling Natural Flavonoid Nanomedicines for Alveolar Macrophage Reprogramming by Restoring Mitochondrial Function in Acute Lung Injury Therapy,” Chemical Engineering Journal 506 (2025): 160171, 10.1016/j.cej.2025.160171.

[42] X. Lin , X. Huang , W. Pi , et al., “Self‐assembly Variation of Glycyrrhetinic Acid Epimers: Assembly Mechanism and Antibacterial Efficacy between 18 α‐GA and 18 β‐GA,” Colloids and Surfaces B: Biointerfaces 242 (2024): 114120, 10.1016/j.colsurfb.2024.114120. 39059147

[43] D. M. Azagury , B. F. Gluck , Y. Harris , et al., “Prediction of Cancer Nanomedicines Self‐Assembled from Meta‐Synergistic Drug Pairs,” Journal of Controlled Release 360 (2023): 418–432, 10.1016/j.jconrel.2023.06.040. 37406821

[44] S. Zheng , W. Wang , J. Aldahdooh , et al., “SynergyFinder Plus: Toward Better Interpretation and Annotation of Drug Combination Screening Datasets,” Genomics, Proteomics & Bioinformatics 20 (2022): 587–596, 10.1016/j.gpb.2022.01.004. PMC9801064 35085776

