# Supplementary material for: A Computational Strategy for Identifying Self‐Assembling Food‐Derived Molecules for Antiparasitic Nanotherapy
Source: Adv Sci (Weinh). 2026 May 29;13(39):e24297. doi: 10.1002/advs.202524297 (PMC13335504; doi:10.1002/advs.202524297)
Supplement: Supplementary file 1 — Supporting File 1: advs75363‐sup‐0001‐SuppMat.docx. [file ADVS-13-e24297-s002.docx]

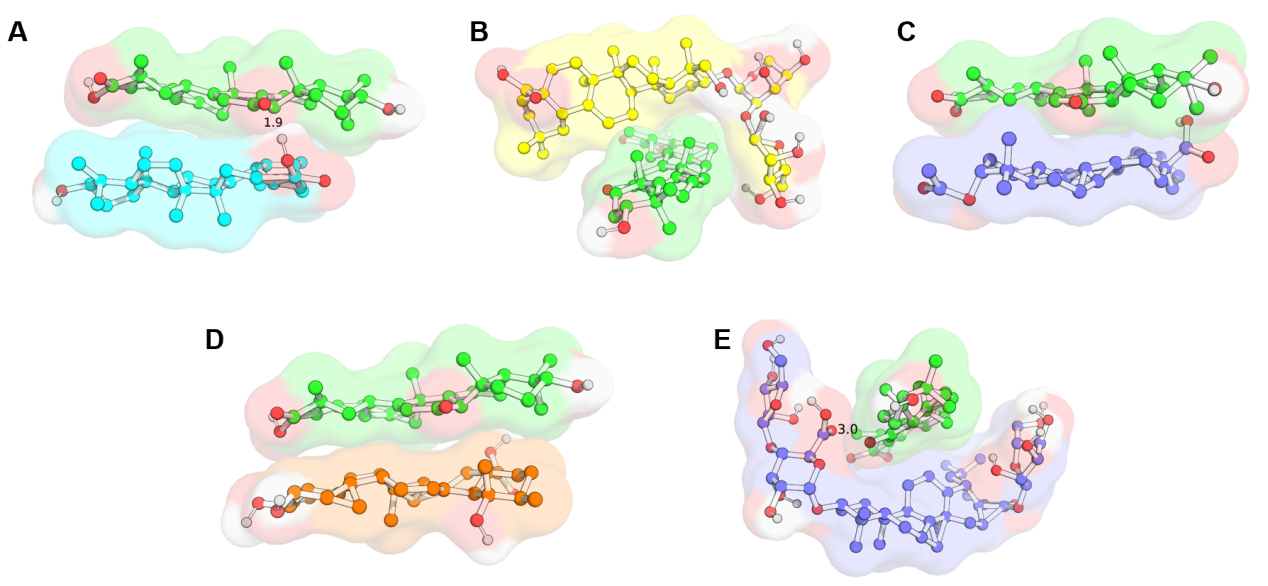


**Figure S1.** Representative molecular docking poses between 18*β*GA and candidate compounds.

(A–E) Representative binding poses of 18*β*GA (green) with (A) PA, (B) CC, (C) UAA, (D) EA, and (E) AA.


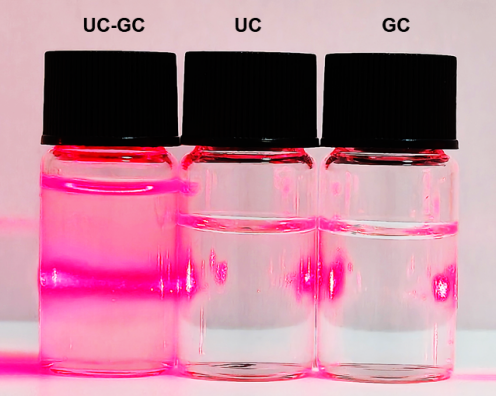


**Figure S2.** Tyndall effect of UA, 18*β*GA, and UA-18*β*GA NP aqueous dispersions.


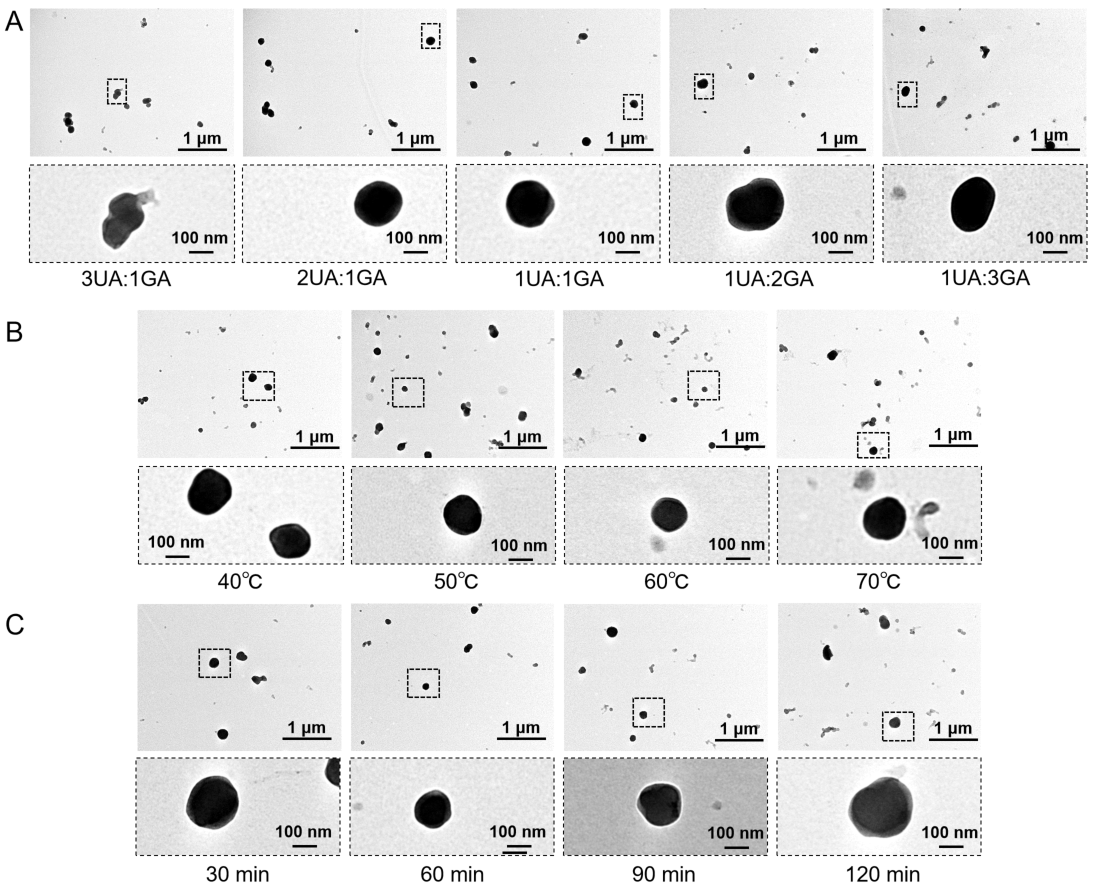


**Figure S3.** Transmission electron microscopy images of UA-18*β*GA NPs prepared under different optimized conditions. (A) UA/18*β*GA molar ratios of 3:1, 2:1, 1:1, 1:2, and 1:3. (B) Preparation temperatures of 40, 50, 60, and 70°C. (C) Preparation times of 30, 60, 90, and 120 min.


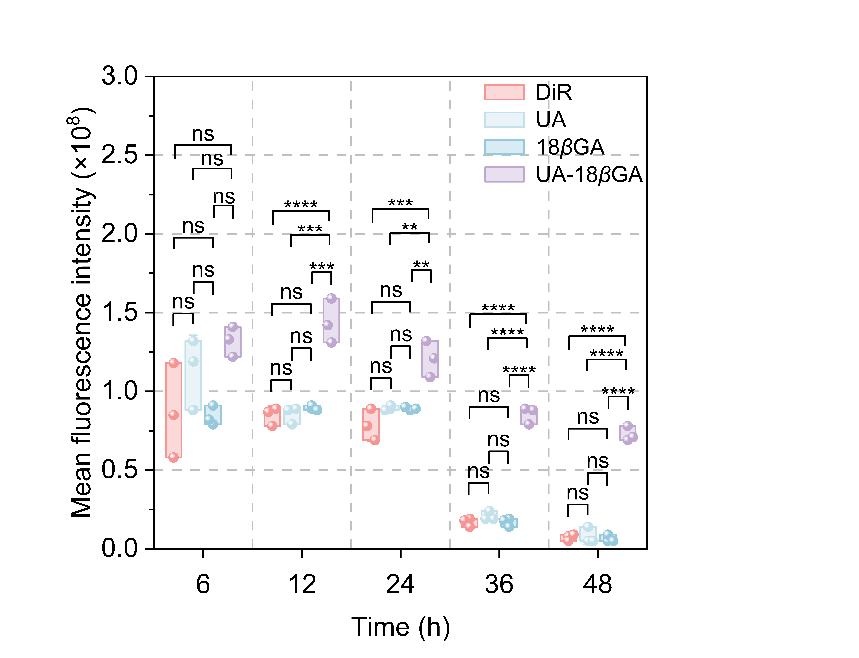


**Figure S4.** Quantification of mean fluorescence intensity from *in vivo* fluorescence imaging of zebrafish after administration of free DiR, DiR-labeled UA, DiR-labeled 18*β*GA, or DiR-labeled UA-18*β*GA NPs.


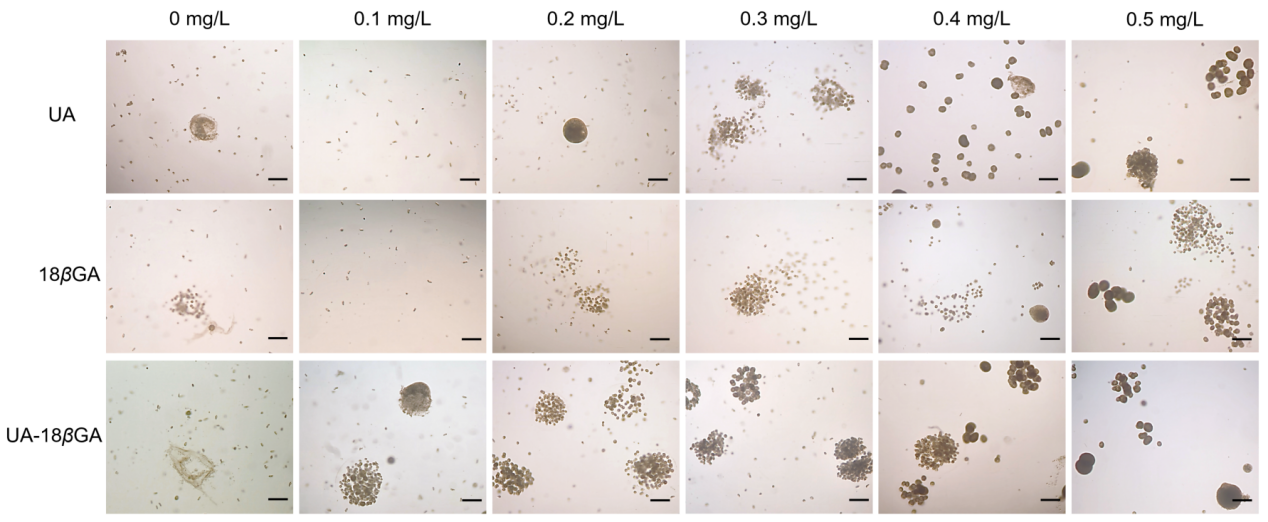


**Figure S5.** Microscopic evaluation of parasite reproductive stages after treatment with UA, 18*β*GA, or UA-18*β*GA NPs (0.1-0.5 mg/L). Scale bar: 500 μm.


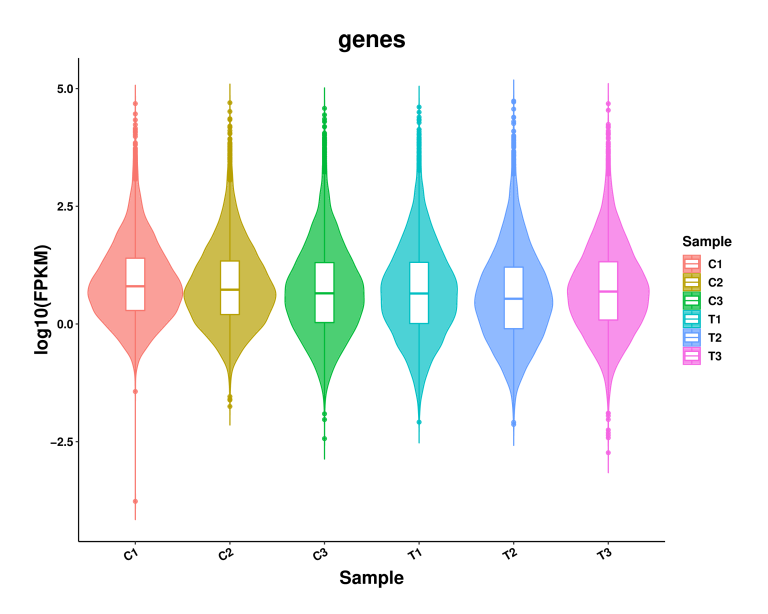


**Figure S6.** Gene expression abundance across samples determined by RNA sequencing.


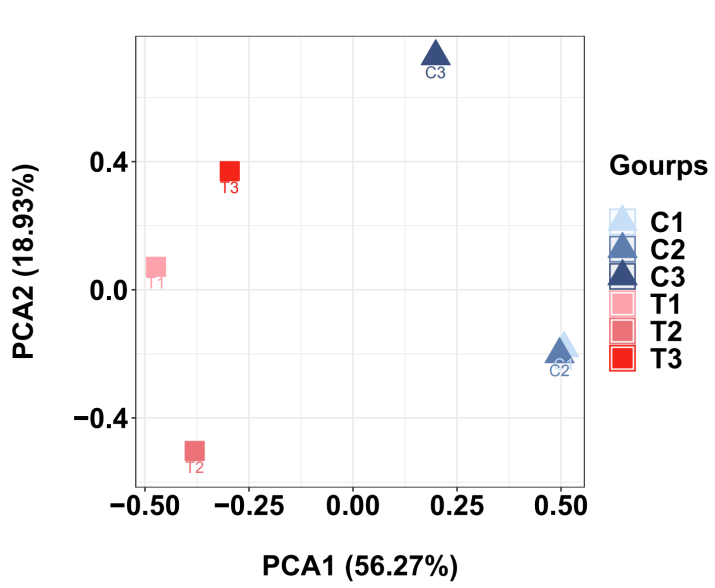


**Figure S7**. Principal component analysis (PCA) of transcriptomes from untreated parasites and parasites treated with 0.5 mg/L UA-18*β*GA NPs.


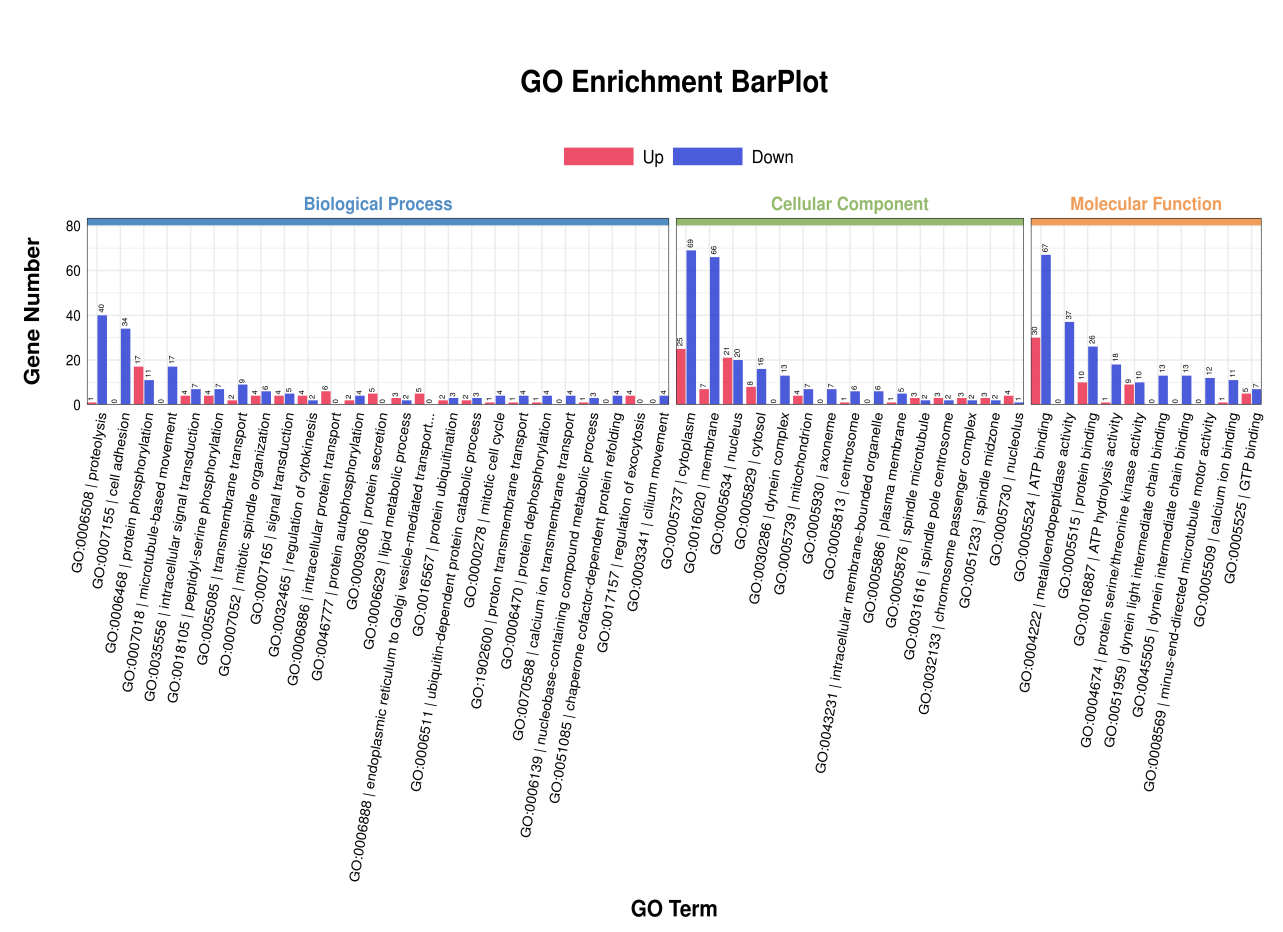


**Figure S8.** Gene Ontology (GO) enrichment analysis of differentially expressed genes (DEGs) between the control and UA-18*β*GA NP-treated groups.


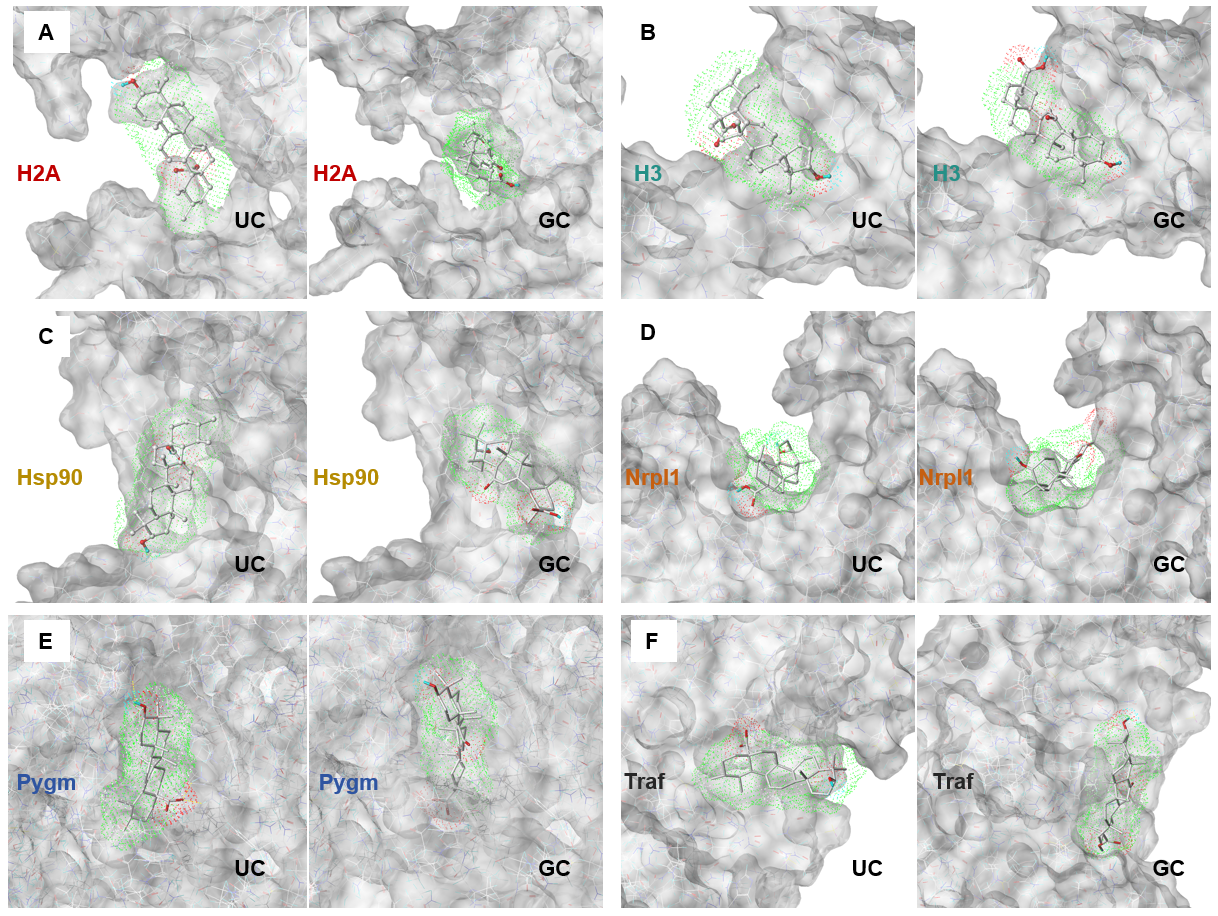


**Figure S9.** Predicted binding poses of UA or 18*β*GA in the active sites of candidate target proteins. (A-F) Predicted binding poses of UA or 18*β*GA in the active pockets of (A) H2A, (B) H3, (C) Hsp90, (D) Nrpl1, (E) Pygm, and (F) Traf.


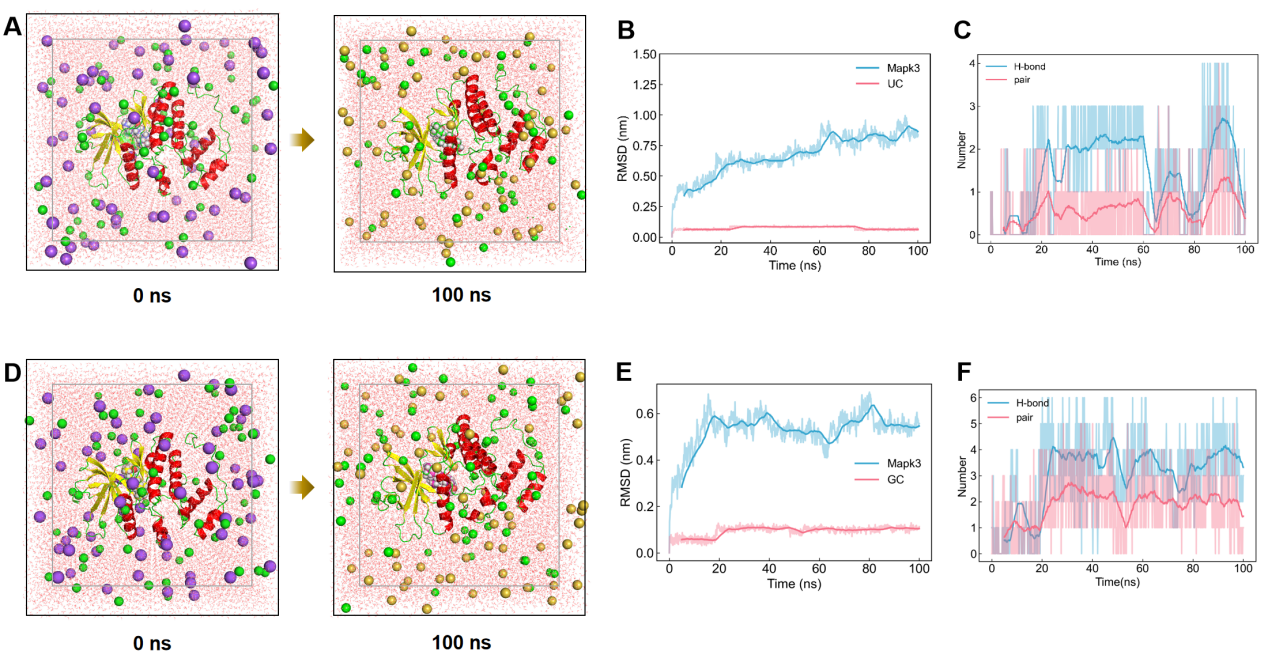


**Figure S10.** Molecular dynamics simulation analysis of the UA-Mapk3 and 18*β*GA-Mapk3 complexes over 100 ns. (A,D) Representative snapshots of the Mapk3 protein in complex with UA (A) or 18*β*GA (D) at 0 and 100 ns. (B,E) Root-mean-square deviation (RMSD) of the UA-Mapk3 (B) and 18*β*GA-Mapk3 (E) complexes during the 100 ns simulation. (C,F) Time-dependent changes in the number of intermolecular hydrogen bonds in the UA-Mapk3 (C) and 18*β*GA-Mapk3 (F) complexes.


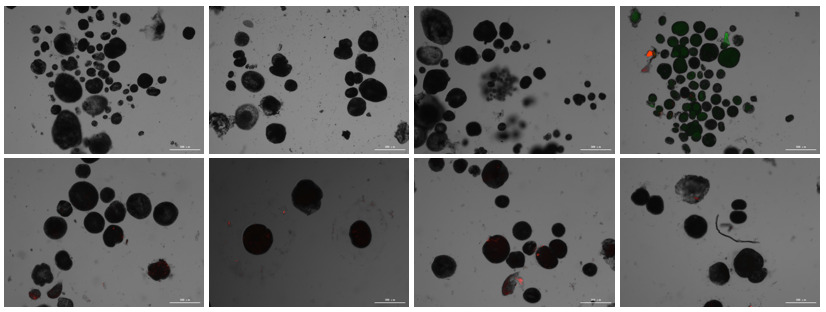


**Figure S11.** Morphological assessment of *I. multifiliis* protomonts under different treatments. Bright-field microscopy images of protomonts treated with (i) different concentrations of UA-18*β*GA NPs and (ii) UA-18*β*GA NPs in combination with the Akt activator SC-79.


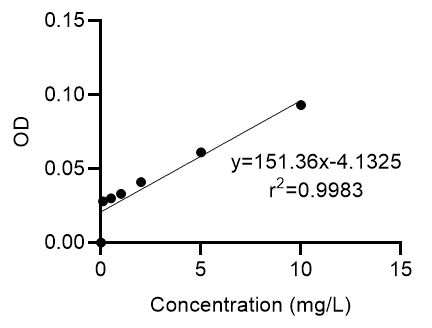


**Figure S12.** Standard curve of Evans blue for quantitative assessment of vascular permeability.

**Table S1.** Primers used for RT-qPCR analysis of toxicity-related genes in zebrafish.

| Accession number | Gene name | Sense | Sequence |
| --- | --- | --- | --- |
| NM_131031.2 | *β-actin* | Forward | AGAGCTATGAGCTGCCTGACG |
|  |  | Reverse | CCGCAAGATTCCATACCCA |
| NM_131562.2 | *baxa* | Forward | GGCTATTTCAACCAGGGTTCC |
|  |  | Reverse | TGCGAATCACCAATGCTGT |
| NM_131846.3 | *ache* | Forward | TTGCTCTTGCCCACTGTGCTACTC |
|  |  | Reverse | CTTCACTCATCACTCTGTTGGGGTTC |
| NM_131879.2 | *cyp1a1* | Forward | CGAAAATCCCAGACGGGCTAC |
|  |  | Reverse | CCCTCATTACTGATGTGCTCCTCT |

**Table S2.** Primers used for RT-qPCR analysis of antioxidant-related genes in zebrafish.

| Accession number | Gene name | Sense | Sequence |
| --- | --- | --- | --- |
| NM_131031.2 | *β-actin* | Forward | AGGTCATCACCATCGGCAAT |
|  |  | Reverse | GATGTCCACGTCGCACTTCA |
| NM_131294.1 | *sod1* | Forward | GGCCAACCGATAGTGTGAGA |
|  |  | Reverse | ACTTTCCTCATTGCCACCCT |
| NM_130912.3 | *cat* | Forward | TATTTCGCGGAGGTTGAGCA |
|  |  | Reverse | GGTAGGAGAAAAGACGCCCC |
| NM_001127516.1 | *ho1* | Forward | AGCAAAAGAGGCAGGAAGCA |
|  |  | Reverse | GCCCACTCCTAATGCGAACT |
| NM_001204272.2 | *nqo1* | Forward | TTTGCAGAATCCCGAGCACT |
|  |  | Reverse | CTTCTGCGATCAAGCTGAAAGA |
| NM_182889.1 | *nrf2* | Forward | AAGCAGACGGAGGAGGAG |
|  |  | Reverse | GGAGGTGTTCAGGCAAGG |

**Table S3.** Primers used for RT-qPCR analysis of inflammation-related genes in zebrafish.

| Accession number | Gene name | Sense | Sequence |
| --- | --- | --- | --- |
| NM_212844.2 | *il1β* | Forward | CCCCAATCCACAGAGTTT |
|  |  | Reverse | TTCACTTCACGCTCTTGG |
| XM_017352586 | *il12* | Forward | CCGAAGGAAAGAGTATCACC |
|  |  | Reverse | CTCAGTTGGGAGCAGTCA |
| NM_212859.2 | *tnfα* | Forward | CATTTGGCTGTGGGCCTTTG |
|  |  | Reverse | TCATCGGGAATGATAATCTC |
| NM_001104937.1 | *nos2a* | Forward | ATCTCCCAGATGCCACTCCT |
|  |  | Reverse | ATCCGCCGCTGTAGAGAGTT |
| NM_001020785.2 | *il10* | Forward | ACGCTTCTTCTTTGCGACTG |
|  |  | Reverse | GAAAGCCCTCCACAAATGAGC |
| NM_182873.1 | *tgfβ* | Forward | ATCTGGGTTGGAAGTGG |
|  |  | Reverse | GTCAAGGATTGCGGGTA |
| NM_213123.1 | *mmp9* | Forward | GTTTCTGGTTCTGGGCACCT |
|  |  | Reverse | TAGCATTGGAGATGACCGCC |
| NM_199611.3 | *arg2* | Forward | TCCAGGCGAGCATGTATTCC |
|  |  | Reverse | TCAAAGCTCAGGTGGATCGG |
| XM_068222147.2 | *nlrp3* | Forward | TCATCTGCTTCTCTCTGACGG |
|  |  | Reverse | CTTTCATACGCCACACCGAC |
| NM_131495.3 | *asc* | Forward | CGCGTCACAAAGTCTGCAAT |
|  |  | Reverse | ATGTGAACACGCCGACCATT |
| NM_001123265.1 | *ikbβ* | Forward | CAAACTGGTGGTTCAAGCCG |
|  |  | Reverse | CACTCACTGGACTGCGAACT |
| NM_131505.2 | *caspasea* | Forward | ATCAAGGACCATCTTCAGGACG |
|  |  | Reverse | AACTCCTCCTTGCCCTGTGT |

**Table S4.** Primers used for RT-qPCR analysis of apoptosis-related genes in *I. multifiliis.*

| Accession number | Gene name | Sense | Sequence |
| --- | --- | --- | --- |
| XM_004039269.1 | *bax1i* | Forward | TTCACTTTTATGTGCCATAGAGCAG |
|  |  | Reverse | AGCCATCATAGCAACATGAGTA |
| XM_004031336.1 | *traub* | Forward | GGGCGAAAATGCATCTAATATCC |
|  |  | Reverse | GAGGAGTCCATCTTATCCTACTTCT |
| XM_004039593.1 | *calpc* | Forward | AATGCCCGGTTTTCCTCCTT |
|  |  | Reverse | GGATGTTGCGAAATCCTCACT |
| XM_004034644.1 | *capn2* | Forward | ACCGATGGTGGATGGAATGG |
|  |  | Reverse | AGGAGGACAAACACCTCCCT |
| XM_004036758.1 | *capn3* | Forward | TAGAAAAAGCCTGGGCAAAGT |
|  |  | Reverse | TGCCCGAAGTCCATAAGCAG |
| XM_004027466.1 | *gpit* | Forward | TGCAGTCCGTGGTTTAGGTG |
|  |  | Reverse | ATCCATCACCTCCATGCCCT |

**Table S5.** Primers used for RT-qPCR analysis of vascular adhesion molecules in mice.

| Accession number | Gene name | Sense | Sequence |
| --- | --- | --- | --- |
| NM_001289726.2 | *gapdh* | Forward | GAAGGGCTCATGACCACAGT |
|  |  | Reverse | GGATGCAGGGATGATGTTCT |
| NM_011693.3 | *vcam1* | Forward | TCTTGGGAGCCTCAACGGTA |
|  |  | Reverse | CAAGTGAGGGCCATGGAGTC |
| NM_010493.3 | *icam1* | Forward | GGACTCACCTGCTGGTCTCT |
|  |  | Reverse | CCCCTGCGATCTAGGAATTT |

**Table S6.** Primers used for RT-qPCR analysis of inflammation-related genes in mice.

| Accession number | Gene name | Sense | Sequence |
| --- | --- | --- | --- |
| NM_008361.4 | *il-1β* | Forward | ACGGACCCCAAAAGATGAAG |
|  |  | Reverse | TTCTCCACAGCCACAATGAG |
| NM_001436390.1 | *inos* | Forward | CTATGGCCGCTTTGATGTGC |
|  |  | Reverse | TTGGGATGCTCCATGGTCAC |
| NM_019388.3 | *cd86* | Forward | GCAGCACGGACTTGAACAAC |
|  |  | Reverse | CCTTTGTAAATGGGCACGGC |
| NM_013693.3 | *tnf-α* | Forward | CTTCTGTCTACTGAACTTCGGG |
|  |  | Reverse | CAGGCTTGTCACTCGAATTTTG |
| NM_010548.2 | *il-10* | Forward | GCTCTTACTGACTGGCATGAG |
|  |  | Reverse | CGCAGCTCTAGGAGCATGTG |
| NM_008625.2 | *cd206* | Forward | TACATGGAGATTGTCAACGTCAG |
|  |  | Reverse | CCAGACCGACTATTGTCTTGC |
| NM_007482.3 | *arg-1* | Forward | GTGAAGAACCCACGGTCTGT |
|  |  | Reverse | AGAAAGGACACAGGTTGCCC |
| NM_011577.2 | *tgf-β* | Forward | AGCTGCGCTTGCAGAGATTA |
|  |  | Reverse | GGGCTGATCCCGTTGATTTC |
| NM_001359638.1 | *nlrp3* | Forward | CAAGGCTGCTATCTGGAGGAAC |
|  |  | Reverse | ATACAGCCTTTCTCGGGCG |
| NM_007700.2 | *ikba* | Forward | TGTGTGGTAACTCCTCAAGATGG |
|  |  | Reverse | GGCTCCAGGACAGTAAACGA |
| NM_023258.4 | *asc* | Forward | GACAGTACCAGGCAGTTCGT |
|  |  | Reverse | AGTCCTTGCAGGTCAGGTTC |
| NM_009807.2 | *capase-1* | Forward | GGACCCTCAAGTTTTGCCCT |
|  |  | Reverse | GCAAGACGTGTACGAGTGGT |
